# Advances in Polyethyleneimine‐Derived Nanoformulations

**DOI:** 10.1002/smsc.70330

**Published:** 2026-07-23

**Authors:** Mohamed S. Attia, Mariusz Skwarczynski, Waleed M. Hussein

**Affiliations:** ^1^ School of Pharmacy and Medical Sciences Griffith University Southport Queensland Australia; ^2^ School of Chemistry and Molecular Biosciences The University of Queensland St Lucia Queensland Australia; ^3^ Monash Institute of Pharmaceutical Sciences Monash University Parkville Victoria Australia

**Keywords:** adjuvant, drug delivery, gene delivery, polyethyleneimine, vaccine delivery

## Abstract

Formulations derived from polyethyleneimine (PEI) serve as versatile and efficient vehicles for the delivery of genes, drugs, and vaccines that are low‐immunogenic and viable alternatives to viral vectors. PEI ensures efficient endosomal escape, preventing the therapeutic cargo from degradation, enhancing uptake, and facilitating effective cytoplasmic release via the proton sponge effect. By combining PEI with tailor‐made delivery vehicles, such as polymeric assemblies, lipid‐based systems, and inorganic nanomaterials, enhanced targeting, safety, and therapeutic efficacy can be accomplished. PEI‐based systems are capable of delivering a wide range of drugs; in particular, they are suited to delivering drugs with a negative charge. A further function of PEI is to activate antigen‐presenting cells and stimulate cytokine production in order to enable the delivery of vaccines. In spite of the promise of PEI‐based formulations, biocompatibility remains a substantial concern. The most effective ways to increase PEI biocompatibility include optimizing charge density, molecular weight, and branching, developing targeted and responsive delivery systems, and using chemical modifications. To pave the way for future clinical applications, we discuss strategies to increase PEI safety, as well as recent advances and prospects in PEI‐based delivery approaches for gene, drug, and vaccine delivery.

## Graphical abstract

This article illustrates the cytotoxic effects of polyethyleneimine (PEI) on cell membranes and mitochondria, the strategies employed to mitigate PEI‐associated toxicity through chemical modification, PEGylation, and structural optimization, and the major types of PEI‐based formulations developed for the delivery of therapeutic drugs, nucleic acids, and vaccines in various biomedical and translational applications.

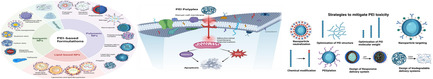

## 1 Introduction

Polyethyleneimine (PEI), a synthetic linear or branched cationic polymer, is characterized by a dense network of primary, secondary, and tertiary amine groups interconnected by short ethyl spacers that contribute to an exceptionally high charge density capable of complexation [1]. Over the past three decades, PEI has emerged as a leading component in nonviral vectors for gene, drug, and vaccine delivery due to its unique advantages, including delivery of sensitive cargo into cells, favorable release profiles, high versatility, and low immunogenicity [2]. A pivotal milestone in the development of PEI as a delivery system was its application as a transfection reagent in 1995 by Boussif et al. [3], who used PEI to transfer DNA into the brains of newborn mice. This breakthrough not only highlighted the potential of PEI as a delivery system but also facilitated its widespread adoption in gene therapy research [4, 5].

It is important to note that complexation alone does not ensure effective delivery, as other factors, like size and charge density, also contribute to the effectiveness of PEI as a delivery system by influencing cellular uptake and subsequent release of nucleic acids and oligonucleotides within the target cells [6]. The structural features of PEI enable it to interact efficiently with negatively charged cargo, such as antibodies, peptides, or small molecules, through covalent or electrostatic interactions. The inherent structural complexity of PEI allows tunability and targeting of specific organs, increasing delivery efficiencies [7]. Production of PEI is also more cost‐effective compared to most other nanocarriers [8].

Gene therapy has emerged as a promising strategy for treating genetic and acquired disorders by controlling the dysregulated expression of specific genes [9]; however, clinical translation of this technology has been impeded by difficulties associated with delivering intact genetic material in vivo [10]. Viral vectors can efficiently deliver genes but are limited by toxicity, immunogenicity, and risk of mutations, prompting research into nonviral delivery systems as safer options [4, 11]. PEI‐based delivery systems are distinguished as nonviral delivery candidates with potential to alleviate some of the key concerns associated with other nonviral vectors. For example, other nanovectors, such as peptide‐ and lipid‐based systems, often lack the stability or specificity needed for targeted applications [9], while PEI binds to genetic material, forming stable polyplexes that protect it from enzymatic degradation and enhancing cellular uptake through cell membrane association [12]. Notably, the stability and efficiency of PEI distinguish it from alternatives like Lipofectamine, calcium phosphate, and electroporation, which frequently fail to meet the demands of in vivo applications [13].

In addition to the fundamental role PEI plays in gene delivery, it is also a versatile biomaterial that contributes to advanced vaccine delivery by acting as both an adjuvant and a carrier to enhance immune responses [7]. Subunit‐based, recombinant protein, and nucleic acid vaccines, such as DNA or RNA‐based vaccines, are among the numerous vaccine forms currently available in modern vaccine technology [14]. Subunit vaccines do not carry the complete pathogen and are considered safer than conventional vaccines owing to reduced risk of disease, allergic reactions, and contamination [15, 16]. However, subunit vaccines are weakly immunogenic and require potent adjuvants to enhance immune responses and delivery efficiency [17]. PEI improves antigen stability and delivery and enhances immune responses, highlighting the vital role PEI plays in vaccine development. PEI promotes synergistic modulation of antigen uptake, activation of antigen‐presenting cells (APCs), and maturation of dendritic cells (DCs), leading to a more robust immune response [18]. In vaccine development, these activities enhance immunity to targeted pathogens or cancer cells stimulated by subunit vaccines.

The unique chemical composition of PEI and its compatibility with diverse ligands secure PEI as a cornerstone in the development of innovative gene, drug, and vaccine delivery systems, making PEI a potential candidate for modern biomedical applications. This review describes the structural and physicochemical properties of PEI, along with its cytotoxicity, and discusses recent advances in PEI‐based formulations for gene, drug, and vaccine delivery.

## 2 Structural and Physicochemical Properties of Polyethyleneimine

**FIGURE 1:**
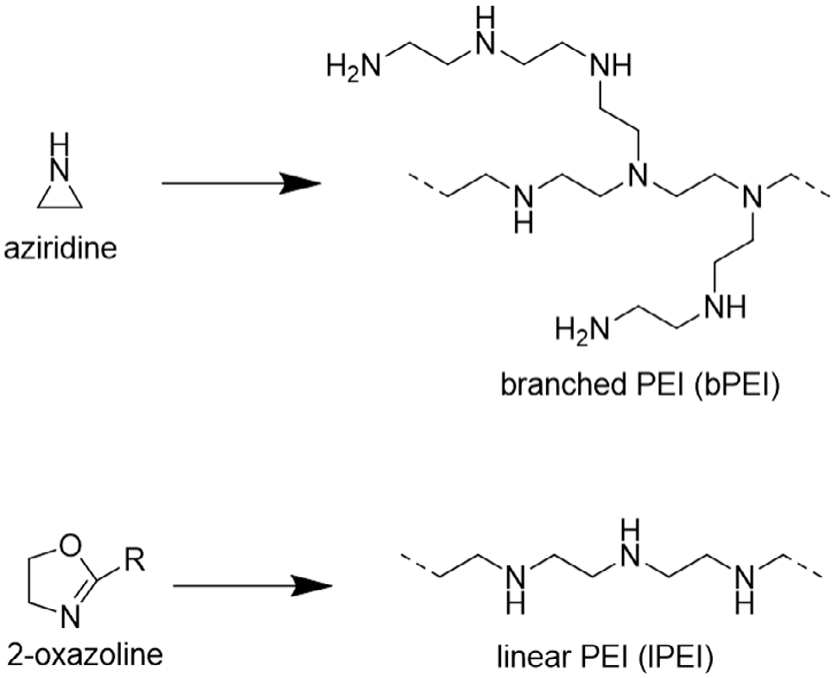
Examples of branched polyethyleneimine (bPEI) and linear polyethyleneimine (lPEI) synthesis.

Polyethyleneimine consists of repeating ethyleneimine units connected via amine groups arranged in two structural forms, linear PEI (lPEI) and branched PEI (bPEI), that exhibit different functional properties (Figure 1) [19]. In bPEI, primary, secondary, and tertiary amines are usually present in approximate ratios of 1:2:1, while lPEI consists of primary and secondary amines that produce more predictable and rigid structures [20]. Branching enhances flexibility and increases the density of amine groups. Primary and secondary PEI amines efficiently bind nucleic acids, making PEI a desirable polymer for delivering genes and drugs [21]. Tertiary amines, although less efficient at binding, exhibit a favorable buffering capacity under acidic conditions, facilitating lysosomal escape through the “proton sponge effect” [7]. In endosomes, PEI becomes highly protonated under acidic endosomal conditions and increases osmotic pressure, causing an influx of chloride ions into vesicles to neutralize the charge. Osmotic pressure continues to increase until endosomal swelling, rupture, and eventual release of the polyplex and cargo into the cytoplasm [22, 23]. Moreover, the strong cationic charge of PEI enhances its solubility and enables robust interactions with negatively charged biomolecules, such as DNA, RNA, and cell membranes [2, 24]. This property allows PEI to condense nucleic acids into stable nanoparticles, contributing to its effectiveness as a nonviral vector for gene therapy. Together, these structural and physicochemical properties have driven research and development of PEI as a transfection agent for sensitive RNA molecules.

Structural differences in lPEI and bPEI arise from the method of synthesis. Polymerization of lPEI is typically synthesized by hydrolyzing oxazolines with acids or bases [25], while bPEI synthesis involves polymerizing aziridine monomers using an acid‐catalyzed ring‐opening method, as depicted in Figure 1. The molecular structure of bPEI is spheroidal, whereas lPEI polymers adopt a double‐stranded helix shape below their melting point (60°C) and transition into trans‐linear chains above their melting point [26]. Viscosity and rheological behavior are important characteristics of PEI influenced by molecular weight, branching, and concentration. For example, higher molecular weight PEI exhibits greater viscosity, which can be beneficial for applications requiring higher stability, although the formulation of higher molecular weight PEI presents challenges [27, 28].

Solubility is another important property of PEI and is influenced by polymer length and branching. The structure of bPEI is less rigid, making it more soluble in water and organic solvents than the linear form [7, 29]. PEI is also highly resistant to thermal and chemical degradation, contributing to its suitability in a wide variety of biomedical and industrial applications; however, to further enhance its solubility and biocompatibility as well as expand its application in formulations that require high stability and dispersion, PEI is often combined with other compounds, such as polyethylene glycol (PEG) [27]. In addition to improving PEI stability under specific conditions, chemical modifications can also address challenges associated with aggregation. The molecular weight and structural characteristics of PEI not only influence its physicochemical properties but also define its biocompatibility, bioactivity, and transfection efficiency, as discussed in the following sections [30].

## 3 Polyethyleneimine Cytotoxicity and Effective Mitigation

**FIGURE 2:**
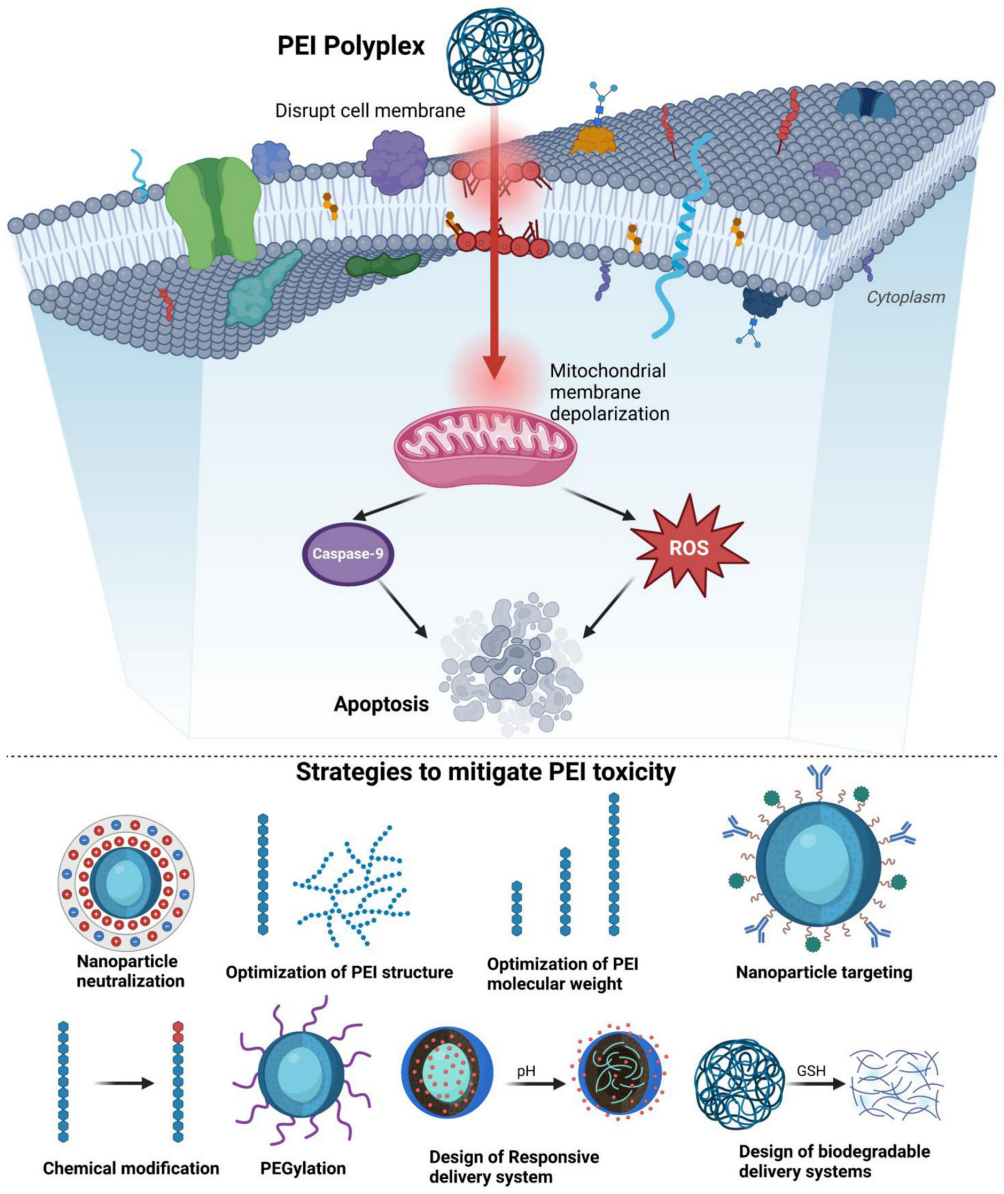
Cytotoxic effect of PEI on cell membranes and mitochondria and strategies to mitigate PEI toxicity. The Figure was designed using Biorender. Figure license number CL27Q9IWZ3.

Polyethyleneimine‐based systems deliver nucleic acids better than most other nanocarrier systems and have shown promise in preclinical settings for carrying therapeutic genes for the treatment of cancer and genetic disorders. However, clinical applications of PEI are limited by cytotoxicity associated with high doses of high‐molecular‐weight PEI and bPEI, which can disrupt membranes and induce inflammatory responses (Table 1) [28]. Understanding the mechanisms involved in PEI‐mediated cytotoxicity is crucial for mitigating these negative effects. First, PEI directly impacts cell membranes by disrupting membrane stability and causing rapid cell damage and decreased viability (Figure 2a) [35]. Subsequently, cytotoxicity occurs within minutes or hours following transfection with PEI, as evidenced by EA.hy926 cell death observed 2 h after exposure to free PEI, likely due to membrane disruption [36]. After cellular uptake, the release of PEI from the complex may trigger intracellular damage through oxidative stress and disruption of lysosomes and mitochondria that can activate apoptotic pathways (Figure 2a) [37] and cell death [38, 39]. Cytotoxicity of PEI can be mitigated by modifying charge density. For example, reduced membrane destabilization and damage have been observed after lowering the cationic charge [40]. Other factors also contribute to PEI cytotoxicity, like pH and membrane curvature, that can affect the surfactant‐like nature of PEI and its role as a proton transfer catalyst [41, 42]. Importantly, PEI cytotoxicity can be mitigated by modifying charge density, molecular weight, and structural branching, and chemical composition, and by adopting other approaches like targeting strategies that increase therapeutic efficacy by using specific ligands, including biodegradable components, and creating controlled release formulations (Figure 2b).

**Table 1:** Comparative evaluation of PEI molecular weights for transfection, cytotoxicity, and biocompatibility.

PEI type	Molecular weight	Transfection efficiency	Cytotoxicity	Biocompatibility	Limitations	Ref.
Low MW PEI	0.6–1.8 kDa	++	+	+++	Poor transfection	[31]
Medium MW PEI	10 kDa	+++	++	++	Requires optimization	[32]
High MW PEI	25 kDa	++++++ (Gold standard)	++++	+	Highly cytotoxic limiting their applicability in vivo	[32, 33]
Branched PEI	25 kDa (branched)	+++++	++++	+	Toxicity, nonbiodegradable	[32]
Modified low MW PEI	1.8 kDa + modifications (Arg, His, PAMAM)	+++++	+	++++	More complex synthesis	[34]

### 3.1 Charge Density and Neutralization Effect

**FIGURE 3:**
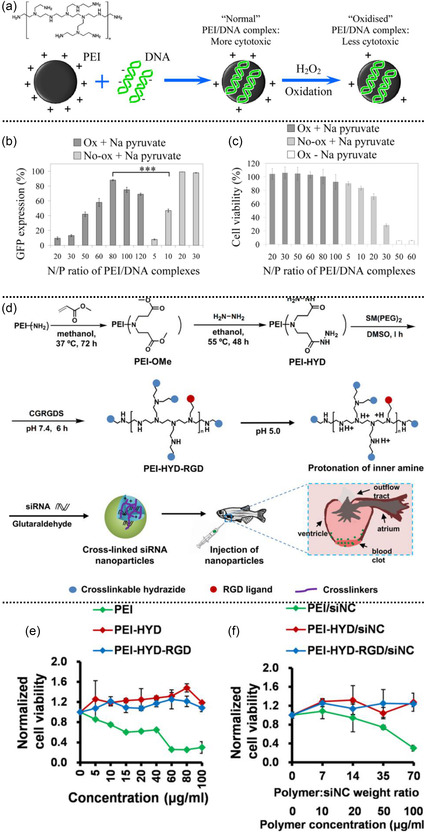
(a) Schematic illustration of the oxidation approach to neutralize the surface charges of PEI/DNA complexes to mitigate their toxicity. (b) Green fluorescent protein (GFP) expression and cytotoxicity of PEI complexes, either with or without oxidation. GFP‐expressing HEK293 cells percentage and (c) AlamarBlue assay shows cell viability for varying N/P ratios of PEI/DNA complexes (**p < 0.001). Adapted with permission [45]. Copyright 2013, American Chemical Society. (d) An overview of the process of synthesizing PEI‐HYD‐RGD materials for assembly of crosslinked nanocomplexes for siRNA delivery to adult zebrafish hearts. (e) Cytotoxicity of PEI, PEI‐HYD, and PEI‐HYD‐RGD at varying concentrations, and (f) nanocomplexes formed at different polymer‐to‐siNC ratios or polymer concentrations. Adapted with permission [46]. Copyright 2016, American Chemical Society.

The high cationic charge density of PEI enhances nucleic acid condensation and endosomal escape; however, this negatively impacts cell viability. Cellular interactions with the strong electrostatic charge of PEI disrupt cell membranes and release enzymes that degrade cells, causing oxidative stress and cell death. While attempting to deacylate commercial 25 kDa PEI, Thomas et al. [43] observed that deacylation increased the charge density and, consequently, the polymer's cytotoxicity [44]. Seow et al. [45] introduced a simple strategy to modify PEI charge density through hydrogen peroxide‐mediated oxidation of amine groups, mitigating cytotoxicity while maintaining transfection efficiency. In this procedure, PEI/DNA complexes were formed, oxidized (Figure 3a), and treated with sodium pyruvate to scavenge hydrogen peroxide radicals that could impair cell viability. Oxidized PEI/DNA complexes exhibited reduced ζ‐potential and increased particle size (due to diminished repulsive forces), while DNA integrity and interactions were preserved. Transfection efficiency of oxidized PEI/DNA complexes in HEK293 cells was 88% at a nitrogen:phosphate (N:P) ratio of 80:1 (Figure 3b). Further, HEK293 cell viability was unaffected (~100%) by oxidized PEI/DNA complexes. In contrast, significant cytotoxicity was observed in response to nonoxidized PEI complexes at N:P ratios greater than 10:1, with ~10% HEK293 cell viability following transfection with nonoxidized PEI complexes at an N:P ratio of 50:1 (Figure 3c).

Hydrazidation can also be used to neutralize PEI charge, reduce cytotoxicity, and improve the effectiveness of PEI as a siRNA delivery system [46]. In a study involving hydrazidation of PEI, primary amines were replaced with hydrazide (HYD) groups, yielding a pH‐sensitive complexation (Figure 3d) that created cross‐linked nanoparticles possessing near‐neutral ζ potentials (±10 mV), lowering the cytotoxicity typically linked to the conventional positively charged PEI systems (+15 to +30 mV). The same study also showed that PEI hydrazides, either combined with arginine‐glycine‐aspartic acid (RGD) or not, did not cause cytotoxicity in HUVEC cells, even at high concentrations up to 100 μg/mL, as opposed to conventional PEI, which was cytotoxic at all experimental concentrations (5–100 μg/mL) (Figure 3e,f). In addition, 0.5 mg/kg PEI‐HYD‐RGD nanoparticles successfully delivered siRNA to zebrafish heart tissues, silencing the aldehyde dehydrogenase 1 family member A2 (Aldh1a2) gene, achieving ~50% gene knockdown.

### 3.2 Molecular Weight and Structural Branching Optimization

The molecular weight of PEI impacts transfection efficiency and cytotoxicity of nanoparticles, with low‐molecular‐weight PEI being less effective at condensing nucleic acids than high‐molecular‐weight PEI. However, higher molecular weight PEI (>25 kDa) increases cytotoxicity by causing membrane disruption and cellular stress. Thus, a PEI molecular weight of 5–25 kDa achieves a balance between efficient nucleic acid delivery and reduced cytotoxicity. Fischer et al. [47] evaluated lactate dehydrogenase (LDH) release from PEI‐ruptured cells and observed that 1616 kDa PEI induced significantly higher cytotoxicity than 12 kDa PEI. Findings showed that 12 kDa PEI produced minimal membrane disruption with no LDH release at 30 or 60 min compared to 1616 kDa PEI that disrupted membranes and elevated LDH levels for 30 min, following 500 µg/mL transfection, and 60 min, following transfection with 5–50 µg/mL.

The degree of PEI branching also increases cytotoxicity. Linear PEIs contain only secondary amines and exhibit significantly lower charge density and less toxicity than bPEI (which comprises primary, secondary, and tertiary amines and has a higher charge density). Furthermore, bPEI causes more damage to cell membranes than lPEI through oxidative stress that triggers the release of intracellular calcium and damages mitochondria.

### 3.3 Chemical Modifications

Chemical modifications can significantly reduce PEI cytotoxicity, especially when combined with biocompatible polymers like PEG. PEGylation of PEI forms a hydrophilic layer to shield positive charges, reducing direct contact with cell membranes and cytotoxicity [27, 48]. Also, PEI‐based formulations are more biocompatible when partially acetylated, further reducing PEI interactions with anionic cellular components. These surface modifications can increase systemic circulation of PEI and PEI‐targeting of cells but can also negatively impact PEI–DNA interaction efficacy [49]. Incorporating targeting ligands alongside PEGylation provides additional safety measures by guiding PEI specifically to target cells, reducing nonspecific interactions and off‐target effects that contribute to cytotoxicity [50]. In a study investigating the administration of PEGylated PEI complexed with transferrin to mice, less toxicity was observed compared to unmodified PEI/DNA, along with twofold higher gene expression within tumors [51].

In a study comparing 0.6 kDa PEI with 25 kDa PEI, Tang et al. [52] found that conjugating β‐cyclodextrin (βCyD) with 0.6 kDa PEI improved transfection efficiency and biocompatibility in human NT2 cells and mouse C17.2 cells, compared to 25 kDa PEI. Cell viability in response to 0.6 kDa PEI/βCyD was 75% and 100% in C17.2 and NT2 cells, respectively, while complete cell death occurred in response to 25 kDa PEI/βCyD. Other PEI modifications include ethylation or acetylation of amines, as well as the addition of negatively charged groups (e.g., propionic or succinic acids). Studying PEI succinylation showed that, compared to unmodified PEI, succinylation reduced toxicity by up to tenfold and achieved an 80% knockdown of the target (luciferase) gene at 50 nM siRNA in Neuro2ALuc cells [53]. Reduced toxicity allows higher polymer concentrations necessary for effectively escaping endosomes.

In a study by Xue et al. [54] fluorination of 25 kDa bPEI with ethyl trifluoroacetate or perfluorobutyryl chloride reduced PEI toxicity. Polyplexes formed at a 50:1 fluorinated PEI:siRNA ratio exhibited 90% siRNA binding affinity. Fluorinated PEI effectively delivered siRNA in vitro (60%–80% knockdown of luciferase in MDA‐MB‐231 cells) and was significantly less toxic than unmodified PEI. Notably, fluorinated PEI shifted the in vivo biodistribution of siRNA nanoparticles from lung accumulation to preferential liver uptake, as shown by ex vivo imaging with Cy5.5‐labeled siRNA injected into mice. Consequently, fluorinated PEI could be applied for liver targeting strategies; however, accumulation of PEI within the liver may exacerbate PEI toxicity and should be further explored.

### 3.4 Targeted and Responsive Delivery

**FIGURE 4:**
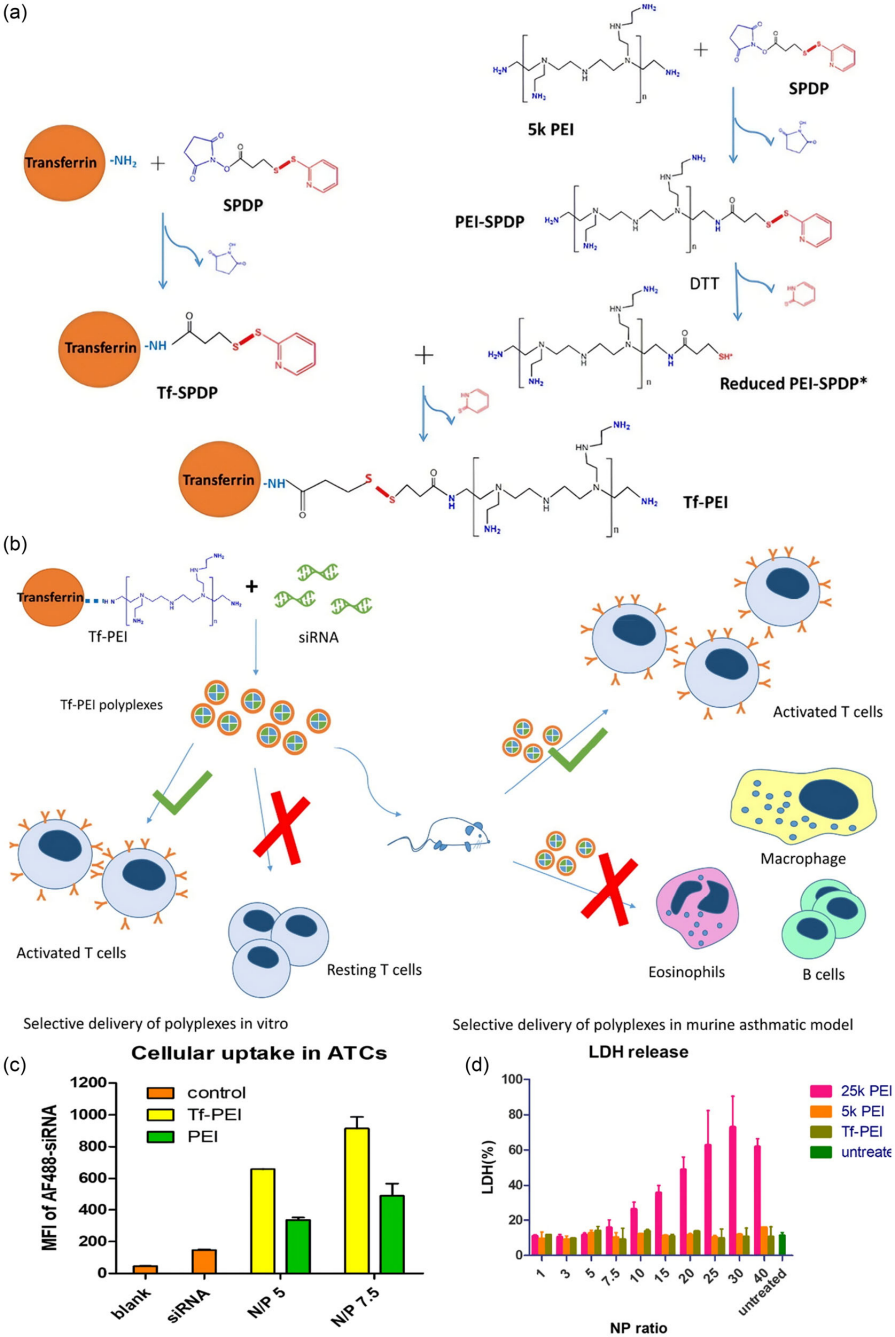
(a) The reaction scheme for the synthesis of transferrin‐coupled Polyethyleneimine (Tf‐PEI) includes the initial reaction between Tf (left) and PEI (right) with N‐succinimidyl 3‐(2‐pyridyldithio)‐propionate (SPDP). Followed by disulfide bond formation between dithiothreitol‐activated PEI‐SPDP and Tf‐SPDP. (b) Tf‐PEI nanoparticles enable targeted delivery of siRNA to activated T cells (ATCs) in asthma, resulting in reduced airway inflammation. (c) Cellular uptake of Tf‐PEI and PEI polyplexes by primary human activated T cells (ATCs) at different N/P ratios. (d) Lactate dehydrogenase (LDH) release from treated A549 cells with Tf‐PEI, 5 kDa PEI, and 25 kDa PEI polyplexes. Adapted with permission [57]. Copyright 2016, Elsevier.

Another approach for mitigating the toxicity of PEI‐based drug and gene delivery systems is to employ targeting strategies that increase therapeutic efficacy using specific ligands, such as antibodies, peptides, or aptamers [2, 55]. By adopting this approach, off‐target toxicity can be reduced due to fewer interactions with healthy tissues. Sun et al. [56] incorporated the RGD peptide into disulfide‐containing PEI/DNA complexes and explored cytotoxicity in 293T and HeLa cells. Disulfide‐containing PEI complexes exhibited lower cytotoxicity than 25 kDa PEI, with cell‐line‐dependent variations observed through higher cytotoxicity in HeLa cells compared to 293T cells. Incorporating RGD did not significantly alter the complex size, which was 100 nm for all complexes. Meanwhile, ζ potential measurements showed a slight decrease in positive charge as RGD content increased (due to the negative charge associated with aspartic acid). In another study involving a mouse model of asthma, PEI polyplexes targeted with transferrin (Figure 4a) significantly reduced toxicity compared to untreated PEI polyplexes, which induced higher secretion of IL‐5 – a pro‐inflammatory cytokine indicative of an inflammatory response (Figure 4b) [57]. The targeting effect of transferrin effectively shields PEI, minimizing its pro‐inflammatory potential, as evidenced by transferrin‐PEI polyplexes showing IL‐5 secretion levels comparable to free siRNA treatments and healthy controls. These modified polyplexes also demonstrated significantly higher receptor‐mediated uptake (Figure 4c) and effective GAPDH gene downregulation compared to PEI polyplexes and were not toxic to lung epithelial cells (A549), even when the N:P ratio was increased (Figure 4d).

Another approach is incorporating PEI into pH‐sensitive delivery systems to exploit the acidic tumor microenvironment (TME), allowing nanoparticles to remain stable under physiological pH conditions and to release their cargo selectively at acidic pH. Additionally, fine‐tuning PEI nanoparticle size could maximize the enhanced permeability and retention (EPR) effect and improve targetability. Sethuraman et al. [58] demonstrated that pH‐sensitive modified PEI polymeric nanoparticles respond differently to physiological pH and the acidic TME, highlighting the potential of these nanoparticles to deliver targeted genes with reduced toxicity. For example, when PEI was complexed with plasmid DNA (pDNA) and poly(methacryloyl sulfadimethoxine)‐block‐polyethylene glycol (PSD‐b‐PEG), nanoparticles with a diameter of ~300 nm were formed and the introduced PEG moiety masked the positive charges of PEI at physiological pH (7.4), resulting in low interaction with cells and a 60% reduction in cytotoxicity in ovarian adenocarcinoma cells (A2780). However, at pH 6.6, the PSD‐b‐PEG complex was shed due to sulfonamide group deionization and hydrophobic aggregation of the complex, allowing PEI/DNA to interact with cells. Consequently, PEG‐deshielding enhanced transfection efficiency but also increased cytotoxicity (as evidenced by a 30% reduction in cell viability). These findings show that PEI cytotoxicity can be reduced under normal physiological conditions with pH‐sensitive formulations that retain high transfection efficiencies. Other stimuli‐sensitive methods, such as local activation by pH or light, can also be employed for tumor‐targeted delivery of PEI and its cargo [59, 60].

### 3.5 Biodegradable and Controlled‐Release Formulations

Several studies have explored biodegradable PEI analogs to mitigate cytotoxicity. Kim et al. [61] synthesized biodegradable polymers by incorporating an acid‐labile imine linker (e.g., glutaraldehyde) into the cytotoxic 25 kDa bPEI. Under acidic conditions replicating the endosomal environment, modified acid‐labile formulations rapidly disintegrated into low‐molecular‐weight PEI. Cell lines treated with modified 25 kDa bPEI exhibited a 35% higher cell viability compared to those treated with unmodified counterparts. Importantly, acid‐labile polymers reduced cytotoxicity associated with PEI without compromising transfection efficiency.

Similarly, low‐molecular‐weight lPEI polymers with disulfide crosslinks delivered nucleic acids more efficiently and without causing cytotoxicity [62]. Disulfide bonds were established between polymers using Lomant's Reagent (LR), and cystine‐based disulfide linkages were established using Boc‐cystine (BC). Disulfide crosslinks conferred biodegradability to the product in the presence of intracellular reducing agents like glutathione, increasing stability and inducing effective activation within the cells. These nanoparticles achieved a balance between effectiveness and biocompatibility by producing 70 ± 4% transfection in HEK cells while maintaining high cell viability (99 ± 5%). In CHO‐K1 cells, these nanoparticles also efficiently delivered DNA by being taken up by >95% of cells within 6 h. Interestingly, the biodegradable PEI formulations were twofold more efficient at transfecting CHO‐K1 cells than commercial reagents, like Lipofectamine and 25 kDa bPEI, by producing ~60% transfection while maintaining >90% cell viability, despite high polymer‐to‐DNA ratios. Even in challenging cell lines such as HeLa, these biodegradable polymers maintained >90% viability.

### 3.6 Systematic Comparative Evaluation of Modification Strategies

Cytotoxicity mitigation of PEI‐based delivery systems often comes at a cost of the delivery efficiency. There is a set of potential trade‐offs associated with each modification strategy outlined in the preceding subsections, ranging from transfection efficiency, biocompatibility, to synthetic and scale‐up feasibility. These limitations, along with the benefits of each approach, were systematically analyzed to demonstrate the steps necessary to steer away from error‐prone empirical trials.

As Table 2 illustrates, optimization of charge neutralization or PEI's molecular weight and branching represents the most accessible entry points to tackle cytotoxicity, as both rely on commercially available materials with scaling up feasibility [63]. Despite the apparent simplicity of these strategies, they tend to penalize their cellular uptake and endosomal escape at a certain threshold of modifications owing to alterations in their cationic nature [47, 64]. Meanwhile, targeted, biodegradable, and stimuli‐responsive formulations sidestep this trade‐off by being conditionally active, retaining their full cationic delivery capacity during systemic and cellular transit. Unleashing their activity is then spatiotemporally controlled via ligand‐directed uptake, stimulus‐triggered deshielding, and/or intracellular backbone cleavage, which together enable efficient cargo release while minimizing off‐target cationic toxicity. The design logic underlying these formulation‐based technologies explains why they consistently strike the optimal balance between efficiency and safety, even though they entail more complicated synthesis and quality control procedures. Thanks to ligand‐mediated targeting, PEI is not chemically modified, but rather the delivery is routed toward the target cells, reducing overall polymer exposure [65]. It is therefore particularly effective in well‐characterized biological environments, where the receptor is overexpressed, whereas this approach is inherently less effective in heterogeneous tissues where the receptor expression is unpredictable [66]. Controlled‐release PEI formulations, on the other hand, do not alter the polymer but rather modulate when and where PEI is delivered to cells [67], yielding formulations conducive to local or sustained delivery, yet upscaling can be made more challenging by the formulation variables. Eventually, modifying PEI chemically produces broad and heterogeneous outcomes, which explains the variability across several domains in Table 2.

**Table 2:** Comparative assessment of PEI modification strategies across key performance, safety, and translational domains.

Strategy	Toxicity reduction mechanism	Transfection efficiency	Biocompatibility	Synthetic complexity	Scalability	Cost	Key limitation	Ref.
Charge neutralization	Reduction of surface cationic charge density	Variable	High	Moderate–Low	High	Moderate–Low	• Requires cargo‐ and architecture‐dependent optimization as efficiency drops sharply beyond optimal neutralization • Polyplex destabilization under physiological conditions	[43, 44, 63]
Molecular weight and structural branching optimization	Use of inherently low‐toxicity PEI with defined architectures	Variable	Variable	Low–Moderate	High (commercially available MW variants)	Low	• Low MW alone is often insufficient for stable polyplex formation	[47, 63, 64]
Chemical modifications	• Steric shielding • Charge masking, • Altered surface chemistry	Variable	Moderate– High	Moderate–High	Variable	Variable (depending on the modification nature)	• PEG dilemma • ABC phenomenon • Detergent‐like toxicity at high lipid ratios	[27, 48, 52, 54]
Targeted and responsive delivery	Dose reduction via receptor‐specific uptake or site‐specific release	High in receptor‐positive cells	High	High	Moderate–Low	High	• Heterogenic efficacy • Requires ligand ratio optimization	[56, 57, 65]
Biodegradable and controlled‐release formulations	Spatiotemporal control of PEI degradation	High	High	Moderate–High	Moderate	High	• Batch‐to‐batch variability concerns • Release profile standardization	[61, 62]

A paradox emerges across PEI modification strategies that perform best biologically, since their scale‐up is undermined by reproducibility concerns and high costs. Nevertheless, those approaches with the greatest scalability, like acetylation, carry functional trade‐offs. Since no strategy possesses overall superiority, these approaches are not mutually exclusive and can be integrated. Among promising PEI platforms proposed to address successive biological barriers are those of combinatorial approaches, for instance, biodegradable PEI cores with cleavable PEG shells with surfaces displaying targeted ligands [68]. Nevertheless, each added process further escalates both the complexity of manufacturing and evaluation, and the combined behavior does not necessarily line up with individual components’ contribution to performance. A choice should therefore be made based on the application perspective rather than a single performance metric. Specifically, ex vivo procedures favor efficiency‐driven approaches such as molecular weight optimization, as embodied by PEIpro (Polyplus‐transfection) [69]. Meanwhile, systemic in vivo delivery calls for more strict clinical‐grade formulation design of more intricate PEGylated and ligand‐targeted systems; whereas controlled‐release formulations are well suited to localized depot administrations.

## 4 Polyethyleneimine‐Based Formulations

**FIGURE 5:**
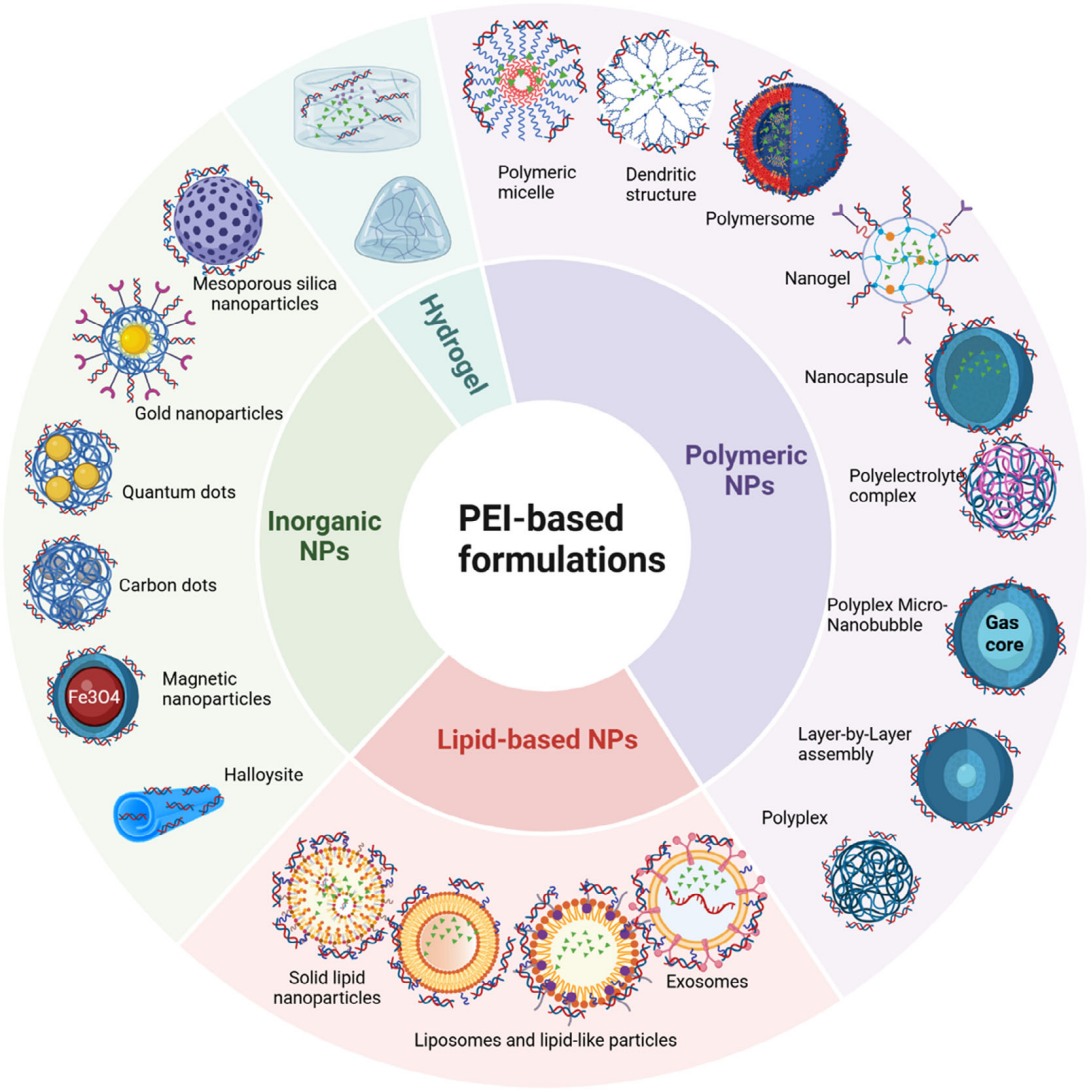
Types of PEI‐based formulations for drug, gene, and vaccine delivery. The Figure was designed using Biorender. Figure license number HH27Q9J63O.

Polyethyleneimine‐based formulations, including polymeric assemblies, hydrogels, inorganic nanoparticles, and lipid‐modified carriers (Figure 5), have shown great promise in gene, drug, and vaccine delivery due to their versatility and tunable properties. Polymeric assemblies like micelles, nanogels, and PEI‐based hydrogels enhance cargo stability and promote gene delivery through electrostatic interactions with nucleic acids. In addition, functionalized inorganic nanoparticles, including mesoporous silica, carbon dots (CDs), and halloysite nanotubes (HNTs), along with lipid‐modified PEI systems, improve cellular uptake, transfection efficiency, and biocompatibility while minimizing cytotoxicity for effective cargo delivery. In addition, modified nanoparticles can penetrate microscopic capillaries, facilitating targeted drug accumulation and promoting sustained drug release [6]. Overall, these PEI‐based formulations aim to provide optimal delivery and biocompatibility while minimizing cytotoxicity.

### 4.1 Polymeric Assemblies

Polyethyleneimine is broadly applied in macromolecule delivery as it efficiently forms stable complexes with negatively charged biomolecules, such as DNA or RNA, through electrostatic interactions. The resultant complexes (polyplexes) shield cargo and promote the transfection of genetic material into targeted cells. Incorporating targeting molecules into PEI polyplexes, such as antibodies or peptides, allows selective targeting of specific cell types or tissues [70, 71, 72]. Polyplexes can also be incorporated into a variety of polymeric assemblies to enhance nanoparticle stability, minimize toxicity, prolong circulation time, and control cargo release while preventing premature degradation.

#### 4.1.1 Polyplex Microbubbles

**FIGURE 6:**
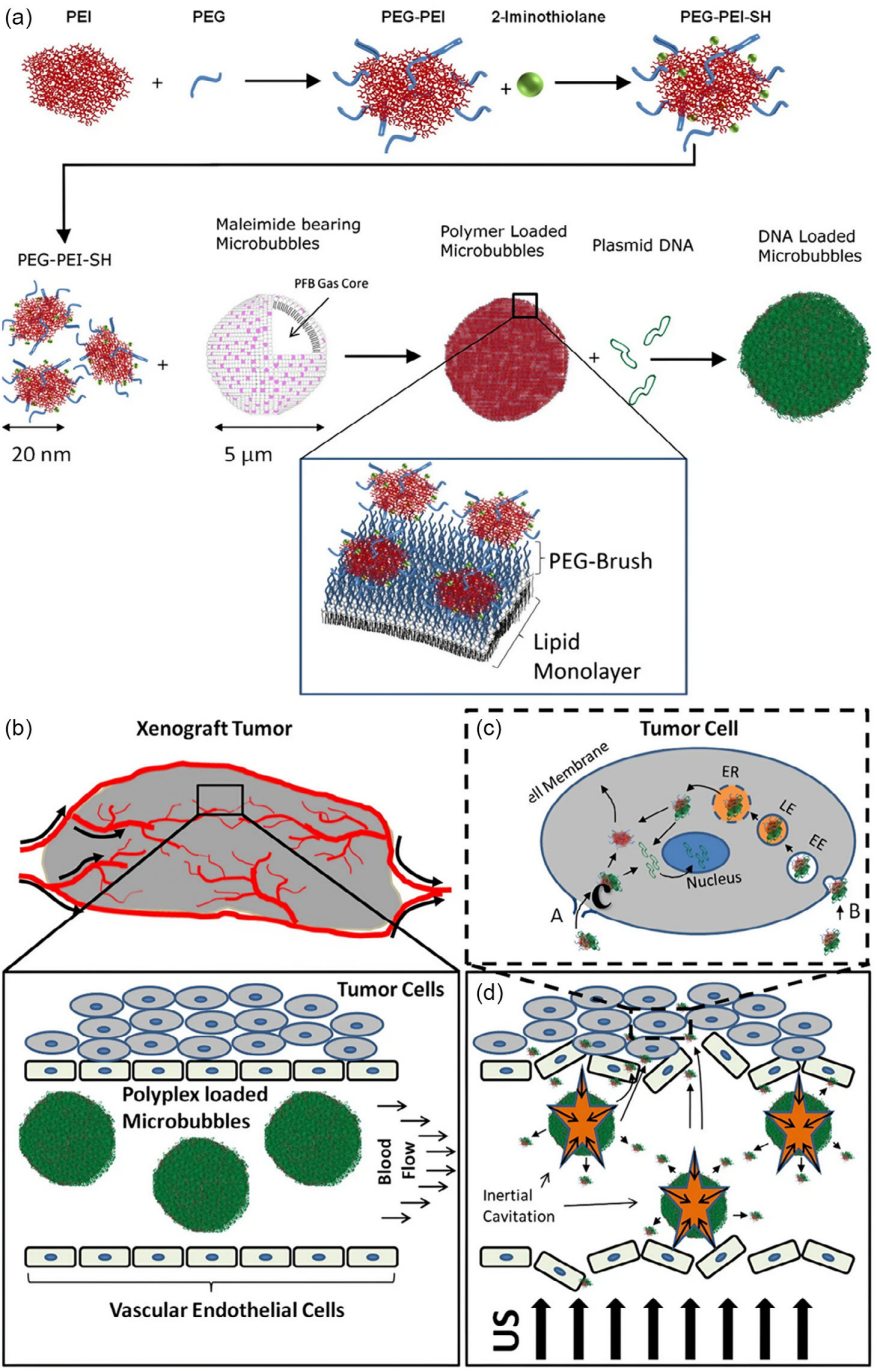
An illustration of polyplex‐microbubble formation and delivery of pDNA to tumor cells. (a) Polyplex microbubbles are produced by covalently attaching thiolated PEG‐PEI to maleimide microbubbles before incubation with pDNA. (b) Systemic intake of polyplex‐microbubbles reaches the tumor vasculature via the bloodstream. (c) Ultrasound is applied to tumor tissue, cavitation occurs, and microbubbles fragment, releasing polyplex/lipid vectors into the tumor tissue. (d) Polyplexes enter tumor cells by either (1) disrupting the physical membrane, allowing passive entry, or (2) facilitating clathrin‐mediated endocytosis, followed by endosomal egress, releasing DNA for nuclear entry and gene expression. Adapted with permission [73]. Copyright 2012, Elsevier.

Polyethyleneimine‐based polyplex‐microbubbles offer an intriguing platform for gene delivery by leveraging the ability of microbubbles to cavitate under ultrasound effect, temporarily altering cell membrane permeability and enhancing intracellular delivery (Figure 6a,c,d). By using polyplex microbubbles, treatments can be delivered with precise spatial and temporal control to achieve optimal efficacy with minimal systemic side effects. Sirsi et al. [73] fabricated PEI‐based polyplex‐microbubbles as ultrasound‐guided gene carriers for in vivo targeted delivery and tumor transfection (Figure 6a). In this study, 25 kDa bPEI‐modified maleimide microbubbles demonstrated a proportional capacity to load DNA relative to the maleimide content. However, higher PEI loading reduced circulation time due to increased nonspecific affinity for blood vessel walls. In vivo testing of ultrasound‐guided delivery confirmed effective transfection of PEI‐microbubbles coupled to a luciferase reporter plasmid in mouse kidneys and produced up to tenfold higher bioluminescence in tumors than untreated tissue. In ex vivo tumor analyses, the ultrasound effect increased gene expression by more than 40 times compared to unexposed control tissues. However, this delivery approach is complicated by reduced circulation time and nonspecific adhesion of microbubbles to the vasculature, reliance on ultrasound for targeting, and microbubble instability – all significant hurdles for clinical translation.

#### 4.1.2 Polyelectrolyte Complexes (PECs)

PECs are formed when positively charged amine groups of PEI interact with negatively charged polyanions (other than cargo) [74, 75]. These complexes provide enhanced stability and bioavailability by protecting therapeutic agents from enzymatic degradation and clearance [76]. Dogaris et al. [77] fabricated PECs as drug delivery vehicles using hemicellulose‐rich lignosulfonate polymers in combination with either PEI or chitosan and agar. The authors noted that the morphology of PECs composed of hemicellulose and PEI varied with composition. For example, PECs at a 2:1 weight ratio (and a 1:1 charge ratio) formed a homogeneous matrix of polyhedral‐shaped spikes (~100 nm face length), while no well‐defined microscopic structures were generated when an excess of hemicellulose (weight ratio 9:1) was applied. As expected, PEI‐based PECs (2:1) were the most stable preparation, with minimal lignin leaching (3.1%–8.3%) and weight loss (≤8.5%). Compared with chitosan‐based PECs, PEI‐based PECs exhibited enhanced stability, reduced weight loss, and hemicellulose leaching, and offered better control over drug loading and release.

Setty et al. [78] modified alginate beads with PEI for controlled furosemide release. Alginate beads synthesized via ionotropic/polyelectrolyte complexation showed high drug loading efficiency (>97%) regardless of formulation parameters, such as calcium chloride concentration, incubation time, or initial drug concentration. However, these beads rapidly swelled, eroded, and released furosemide within 2.5 h in simulated intestinal fluid (pH 6.8), indicating that sustained drug delivery would be unlikely. To address this degradation, alginate beads were coated with PEI to form a polyelectrolyte complex, which created a physical barrier that reduced swelling and erosion of beads while extending drug release time. Increased PEI concentrations and prolonged exposure time directly affected membrane thickness, resulting in thicker coatings that further inhibited drug release.

#### 4.1.3 Nanocapsules

Polymeric nanocapsules are a class of nanocarriers distinguished by their core–shell structure, where active ingredients are enclosed within a polymeric shell [79]. This unique architecture offers several benefits, including protection from environmental degradation and controlled, sustained release [80, 81]. Polymeric nanocapsules range in size from 10 to 200 nm, depending on whether they are made from natural or synthetic polymers, granting them a high level of design flexibility. Lee et al. [82] developed poloxamer/PEI shell‐crosslinked nanocapsules to encapsulate magnetite nanocrystals as a vector for siRNA delivery. Incorporating PEI within the polymeric shell improved nanocapsule stability through cationic charge repulsion. The PEI‐functionalized magnetic nanocapsules significantly increased uptake by PC‐3 prostate cancer cells under an external magnetic field, facilitated by strong electrostatic interactions that promoted cellular internalization and subsequently endosomal escape. The nanocapsules also significantly reduced green fluorescent protein (GFP) expression (by 64%) in MDA‐MB‐435 breast cancer cells, exhibiting greater efficacy than 25 kDa PEI while producing negligible cytotoxicity.

#### 4.1.4 Polymersomes

**FIGURE 7:**
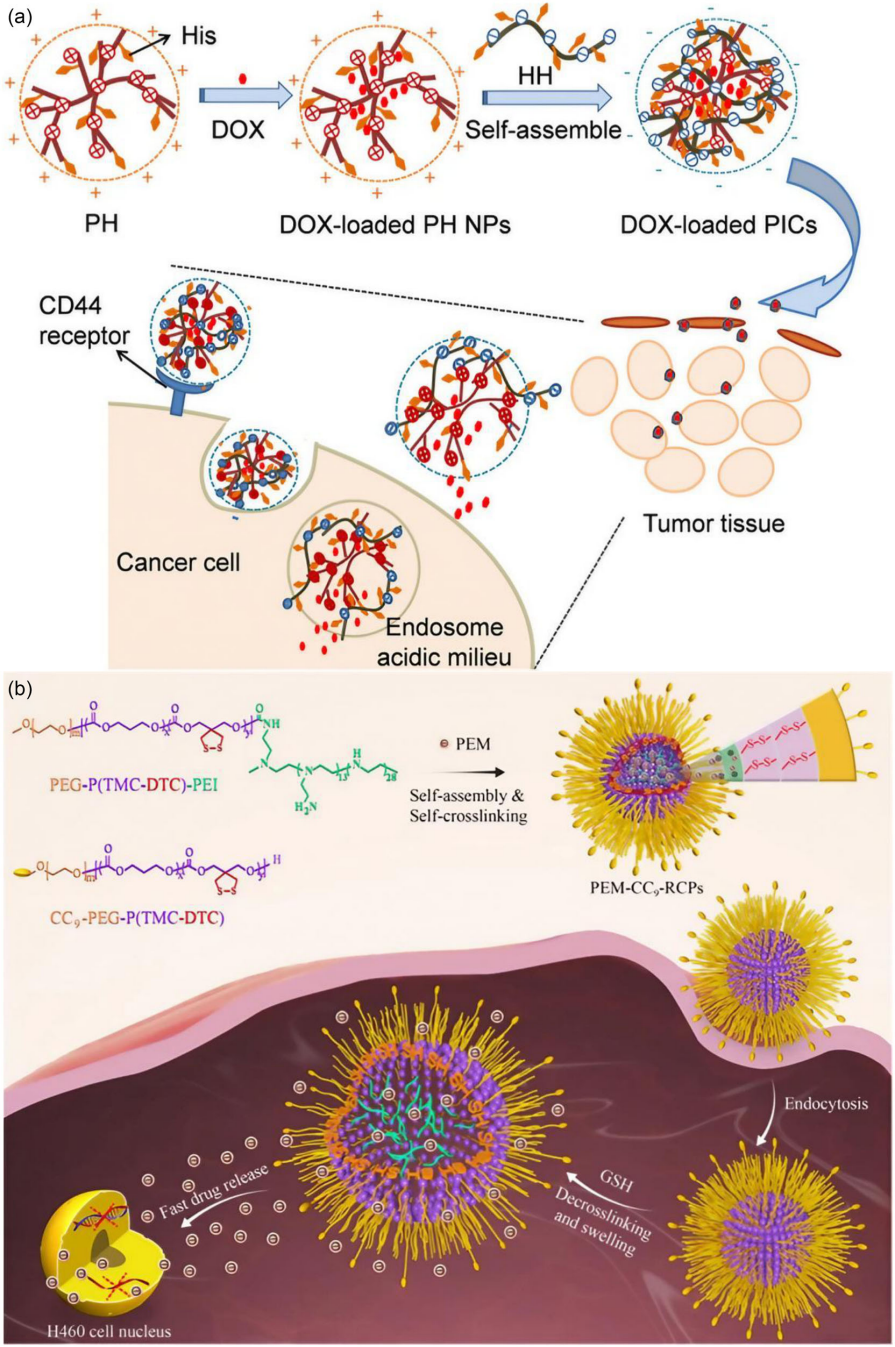
(a) Schematic showing assembly of doxorubicin‐loaded hyaluronic acid‐histidine/PEI‐histidine polyion complexes (HH/PH PICs) along with their features of specific delivery and pH‐responsive release. Adapted with permission [74]. Copyright 2015, Elsevier. (b) Schematic showing the structure and functions of CSNIDARAC (CC_9_) peptide‐functionalized reduction‐responsive chimeric polymersomes (RCPs) for targeted delivery of pemetrexed (PEM) to H460 cells. Adapted with permission [87]. Copyright 2018, Elsevier.

Polymersomes are biomimetic self‐assembling vesicle‐like structures composed of amphiphilic block copolymers that can be tailored for gene or drug delivery [83, 84]. These nanoscale carriers have a hydrophilic core and a hydrophobic bilayer membrane that resembles the liposome structure. However, mechanical robustness makes polymersomes more stable, controllable, and viable for diverse biomedical applications as they can encapsulate hydrophilic and hydrophobic therapeutic agents [85, 86]. Yang et al. [87] developed PEI‐based redox‐responsive chimeric polymersomes (RCPs) functionalized with the CC9 peptide for targeted delivery of pemetrexed disodium in lung cancer therapy (Figure 7b). In this formulation, PEI significantly improved pemetrexed loading efficiency (14%), endosomal escape, and tumor‐targeted delivery. In reductive environments, glutathione triggered pemetrexed release from CC9‐RCPs and increased cytotoxicity by 2.6‐ and tenfold in H460 lung cancer cells compared to nontargeted RCPs and free pemetrexed, respectively. In vivo, the targeted polymersomes circulated 22 times longer and accumulated 9.1‐fold higher in tumor tissue than the clinical formulation Alimta, effectively suppressed H460 xenograft tumor growth, and significantly extended mouse survival compared to nonspecific polymersomes and Alimta.

#### 4.1.5 Dendrimers

Dendrimers are highly branched polymers with tree‐like, uniform, and well‐defined structures [88]. Dendritic nanostructures possess a large surface area, making them excellent for binding multifunctional groups, therapeutic agents, or targeting ligands [89]. Dendrimers are multivalent with tunable chemical properties and tailored surface modifications that contribute to their potential as pharmaceutical carriers, imaging agents, diagnostic agents, and precise organ‐targeting agents [90, 91]. Nam et al. [92] developed bioreducible PEI‐thiol‐modified dendritic derivatives for efficient gene delivery with reduced cytotoxicity. In this study, PEI‐based dendrimers were synthesized using Michael addition and amidation reactions to create disulfide‐containing core molecules that were conjugated with 1.8 kDa bPEI to produce bioreducible dendrimers with varying molecular weights that were biodegradable in the presence of reducing agents like dithiothreitol. These reducible dendrimers formed smaller polyplexes, especially the 16 kDa PEI derivative (61 nm), compared to typical 25 kDa bPEI polyplexes (91 nm), further enhancing gene condensation and delivery capabilities. Among the synthesized dendrimers, the bioreducible 16 kDa PEI derivative formed stable polyplexes with *a* +34 mV ζ potential and exhibited superior transfection efficiency and reduced cytotoxicity in comparison to 25 kDa bPEI, Lipofectamine 2000, and FuGENE 6. In HeLa cells, the transfection efficiency of the bioreducible 16 kDa PEI was 3.6‐fold higher than 25 kDa bPEI and 7.4‐fold higher than Lipofectamine 2000. Importantly, PEI‐based dendrimers produced higher cell viability (>80%) compared to 25 kDa bPEI (<20%).

**FIGURE 8:**
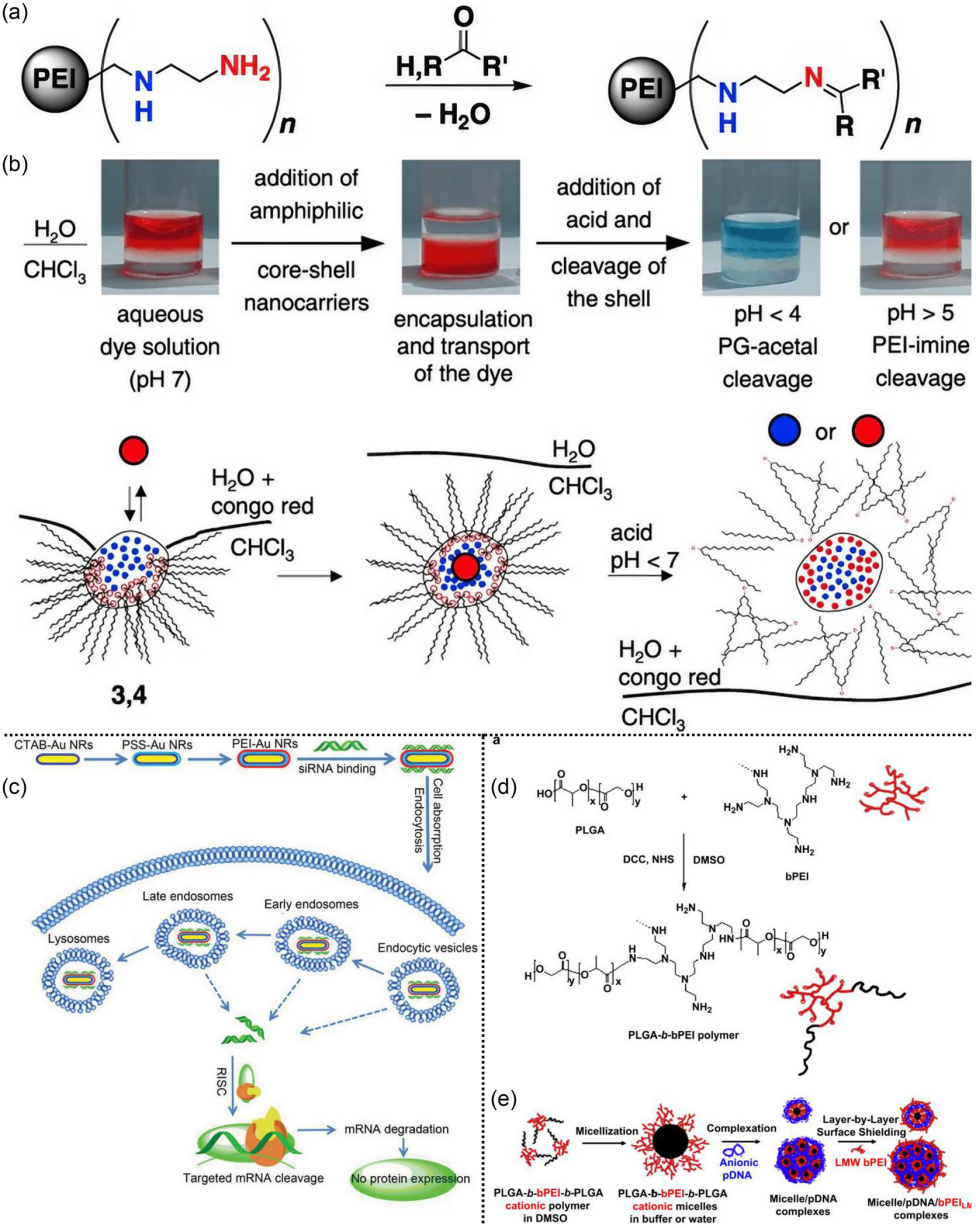
(a) Synthesis of amphiphilic core–shell architectures with PEI's NH_2_ terminals, where NH_2_ groups (red) were functionalized via imine condensation, leaving unreacted linear units (blue). PTSA = p‐toluene sulfonic acid. (b) Encapsulation and transport of polar guest molecules by dendritic nanocarriers (3 and 4) into the organic phase. Upon acidification, the shell cleavage releases the guest into the aqueous phase, as evident with Congo red (pH 4–5). Adapted with permission [93]. Copyright 2002, Wiley‐VCH. (c) An overview of PEI–Au nanorods (NRs) fabrication and application in siRNA delivery. Adapted with permission [94]. Copyright 2014, Wiley‐VCH. (d) Synthetic scheme for 36 kDa poly(lactic‐co‐glycolic acid) (PLGA)−25 kDa bPEI copolymer. (e) Micellization of 36 kDa PLGA‐25 kDa bPEI copolymer, forming micelle/pDNA complexes, followed by LbL surface shielding with bPEI_LMW_. Adapted with permission [95]. Copyright 2011, Elsevier.

Haag and coworkers [93] designed hyperbranched PEI (HPEI)‐functionalized nanoscale dendritic core–shell structures capable of encapsulating cargo (Congo red as a model) and releasing them when exposed to an acidic environment associated with the TME and tissue infection (Figure 8a,b). Carbonyl compounds were condensed between terminal groups of PEI when the core–shell architecture was created, and imine linkages were formed between PEI and aldehydes and ketones, such as 6‐undecanone and 1‐hexadecanal. Since imine bonds are acid‐sensitive, selective cargo can be released in mildly acidic environments (pH 5–6) without compromising system stability at neutral pH (Figure 8b). Encapsulation and release properties were also influenced by molecular weight, degree of alkylation, and core–shell density, with higher functionalization densities (~45%–50%) significantly enhancing the transport capacity of encapsulated cargo.

#### 4.1.6 Layer‐by‐Layer (LbL) Assembly

The LbL assembly technique, often employed to create nanostructured materials by sequentially depositing oppositely charged nanoparticles or molecules [96], is an adaptable and simple process that produces materials with tailored properties and wide applications in drug delivery and tissue engineering [97, 98, 99]. By controlling the composition, thickness, and functionality of layers, the LbL technique can precisely fabricate multifunctional materials [100]. Wang et al. [101] used LbL assembly to deliver genes and developed upconversion nanoparticles (UCNPs) made of sodium yttrium fluoride and erbium modified with layers of PEI and citric acid to reduce PEI toxicity. Strong DNA binding was achieved at ratios of N:P ≥ 20 (according to gel electrophoresis), and the UCNPs exhibited high crystallinity, stable surface charges, and enhanced dispersion in water while maintaining upconversion luminescence emission under 980 nm excitation. Double‐layered coated UCNPs were relatively safe compared to their monolayered counterparts (with cell viability >80% at 0–125 µg/mL) and achieved a transfection efficiency higher than monolayered UCNPs and pure pDNA in HeLa cells, indicating their potential for theranostic applications.

Shen et al. [94] used LbL assembly to deliver siRNA to breast cancer cells by developing functionalized gold nanorods (AuNRs) with PEI. In the LbL process, polystyrene sulfate and PEI were sequentially deposited onto the surface of cetyltrimethylammonium bromide‐stabilized AuNRs (Figure 8c), achieving strong electrostatic interactions with negatively charged siRNA and forming a stable nanocomplex that prevented degradation and facilitated siRNA delivery. Flow cytometry and confocal microscopy demonstrated successful internalization of PEI‐AuNR/siRNA complexes by MDA‐MB‐231 and SUM‐159 breast cancer cells. The delivery system also effectively reduced targeted mRNA levels by 77%.

#### 4.1.7 Micelles

Polymeric micelles typically form when amphiphilic polymers self‐assemble in an aqueous environment [102, 103]. Micelles derived from PEI are nanoscale carriers widely recognized for their unique structure that consists of a hydrophobic core surrounded by a hydrophilic PEI shell [104]. The charge and surface functionality of these nanocarriers are tunable, facilitating controlled release and targeted delivery [105]. Sawant et al. [106] evaluated the effectiveness of dioleoylphosphatidylethanolamine (PE)‐modified PEI as a transfection nanocarrier in B16‐F10 melanoma cells. PEI‐PE/pDNA complexes displayed a critical micelle concentration of 34 μg/mL and, at an N:P ratio of 16:1, exhibited a particle size of 225 nm and a positive surface charge of +31 mV. For enhanced biocompatibility, PEI complexes were modified with PEG‐PE to neutralize the positive charge of nanoparticles that resisted salt‐induced aggregation but retained transfection efficiency in serum‐containing media. The PEI‐PE/pEGFP system also induced higher protein expression compared to 1.8 kDa PEI and 25 kDa PEI complexes, while the addition of PEG‐hydrazone‐PEI created a pH‐responsive nanoplatform sensitive to the acidic TME.

Similarly, Mishra et al. [95] conjugated 36 kDa poly(lactic‐co‐glycolic acid) (PLGA) to 25 kDa bPEI and self‐assembled the conjugate into cationic micelles for pDNA delivery (Figure 8d,e). This delivery system exhibited robust reconstitution properties that conferred micelle/pDNA complexes with stable particle sizes (100–150 nm) and positive surface charges (+30–40 mV). A minimal amount of 2.5 μg bPEI_1.8kDa_ per 1 μg pDNA was added to this delivery system with no particle size changes; however, a positive impact on pDNA shielding was observed, and the transfection efficiency of the reconstituted 1.8 kDa bPEI‐modified system was 16‐fold higher than the basic one and 39‐fold higher than the reconstituted 25 kDa bPEI/pDNA. The PEI‐tuned pDNA–micelle complex also produced minimal cytotoxicity against MCF‐7 breast cancer cells with cell viability comparable to the control, even at high pDNA doses up to 20 μg.

#### 4.1.8 Nanogels

Nanogels, or protective barriers created by cross‐linking polymer networks, protect therapeutic payloads from the external environment. Nanogels are typically controlled by their molecular interactions and mesh networks, which use electrostatic and covalent interactions to precisely release drugs locally [107, 108], reducing the risk of off‐target side effects. Nanogel‐based nanoplatforms have been shown to reduce drug clearance rates and prolong circulation time, enhancing drug retention [109]. Nanogels can also encapsulate hydrophilic and hydrophobic drugs within the same system through various methods, such as covalent conjugation, physical encapsulation, and controlled self‐assembly. Fine‐tuning and modification of nanogels enable drug release in response to specific stimuli in the microenvironment at the target site, such as temperature, pH, enzymes, or external stimuli, through mechanisms like structural degradation, diffusion, or displacement by multivalent ions [110].

Vinogradov et al. [111] developed a cytotoxic agent by crosslinking 25 kDa PEI‐based cationic nanogels loaded with nucleoside analog 5′‐triphosphates. These nanogels were conjugated with 5‐fluoroadenosine arabinoside to facilitate spontaneous ionic interactions between the triphosphate group of the drug and the protonated PEI chains. Subsequently, the nanogels were compacted and enveloped by a hydrophilic 8 kDa PEG, providing steric stabilization and enhanced biocompatibility, while PEI's amino groups enabled efficient conjugation with α‐ or γ‐carboxyl groups of folate moieties. Nanogels carried up to 33% of the drug payload, with ~35% released within 24 h and the remaining medication slowly released over 4 days. In vitro transcellular transport of folate‐nanogel polyplexes into Caco‐2 cells was fourfold higher than the transport of the free drug.

Dissociable lPEI nanogels were also developed to deliver therapeutic thiol‐terminated GFP and vascular endothelial growth factor (VEGF) siRNA into tumor cells [112]. Disulfide crosslinking enabled controlled siRNA release under reductive conditions, positioning the nanogels as promising siRNA delivery vehicles due to their robust crosslinking, stability, and effective gene silencing. Biodegradation of crosslinked lPEI/siRNA nanogels yielded low‐toxicity oligomeric 2.5 kDa lPEI, producing 80% cell viability at an N:P ratio of 60:1. The nanogel (~215 nm diameter and +35 mV) improved cellular uptake, endosomal escape, and siRNA release under reductive conditions, achieving 54% GFP expression reduction and 60% VEGF mRNA silencing.

### 4.2 Polyethyleneimine‐Modified Hydrogels

Hydrogels are 3D polymeric networks composed of hydrophilic polymers that retain large quantities of water and maintain structural integrity without dissolving during the process. Hydrogels are becoming increasingly popular as transdermal patches, injectable gels, and targeted therapies for cancer therapy, wound healing, and tissue engineering due to their versatility, biocompatibility, stimuli‐responsiveness, and controlled release. By using reversible borate ester bonds, polyvinyl alcohol (PVA), and PEI, Wu et al. [113] prepared a dual‐crosslinked hydrogel that responded to pH changes via dynamic borate ester bonds and protonation of PEI amine groups. Loaded with doxorubicin, this hydrogel exhibited a cumulative doxorubicin release of 65% following 24 h of drug release at acidic pH, mimicking the TME, compared to 49% at physiological pH. As pH increased, compressive stress and modulus also increased, indicating an increase in mechanical strength. Over 2 weeks, the hydrogel degraded more slowly in acidic environments compared to neutral and basic environments. Biocompatibility testing of the hydrogel showed high cell viability in NIH/3T3 fibroblasts (86% after 24 h, increasing to 91% after 72 h).

### 4.3 Polyethyleneimine‐Modified Lipid Nanoparticles

Lipid‐based nanoparticles, including liposomes, solid lipid nanoparticles (SLNs), and exosomes, have similar biomimetic properties to biological membranes, making them ideal candidates for advanced drug and gene delivery applications [114, 115]. Various therapeutic payloads can be encapsulated in liposomes, which are spherical vesicles with an internal lipid bilayer [116, 117]. For effective gene delivery, PEI has been incorporated into liposomal structures to increase loading efficiency, stability, cellular uptake, and endosomal escape. Dai et al. [118] from Stephenson group prepared liposomes with PEI to deliver a lipopeptide‐based vaccine (LCP‐1) against Group A Streptococcus (GAS). The vaccine formulations were administered intranasally to outbred mice, eliciting mucosal and systemic immune responses (IgA and IgG, respectively), which effectively opsonized multiple clinically isolated GAS strains.

**FIGURE 9:**
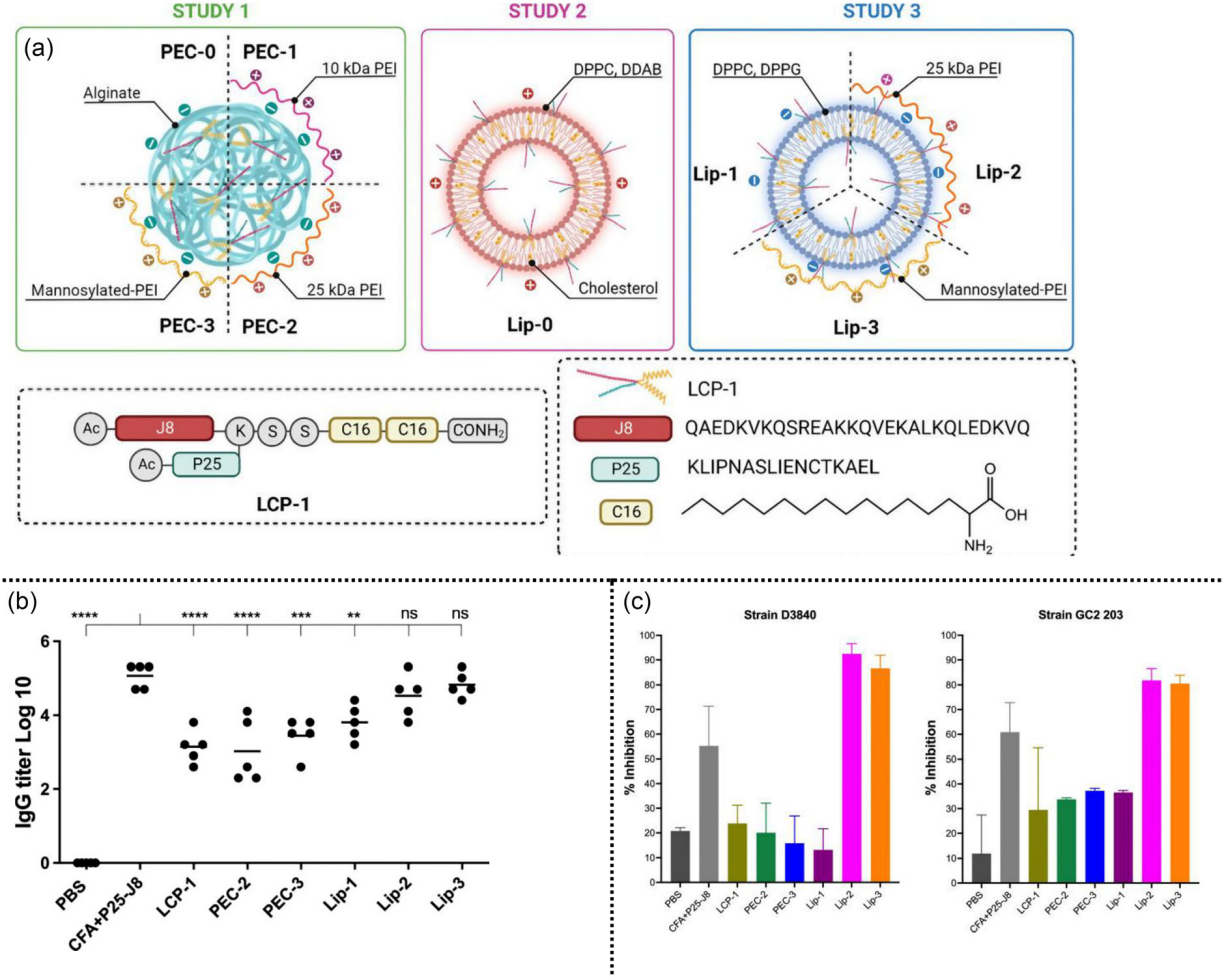
(a) Schematic showing the structure of PECs and liposomes developed for vaccine delivery. (b) J8‐specific serum IgG titers in inbred female BALB/c mice following the first subcutaneous boost. Where, ns: *p* > 0.05; *p < 0.05; **p < 0.01; ***p < 0.001; ****p < 0.0001. (c) Opsonization activity of sera collected from cardiac blood of mice (day 63) against GAS clinical isolates D3840 and GC2 203. Adapted with permission [120]. Copyright 2023, American Chemical Society.

Moreover, Dai et al. [119] also investigated how PEI molecular weight and concentration affected liposomal GAS vaccine adjuvant activity by using different amounts of PEI with varying molecular weights (i.e., 0.6, 1.8, 10, and 25 kDa) to formulate liposomes. Outbred mice intranasally treated with higher ratios of 0.6 kDa PEI in PEI‐to‐lipid content ratios (1:3) produced a stronger immune response than mice treated with lower ratios (1:6 and 1:9). However, PEI molecular weight did not significantly impact adjuvant properties. Hussein and colleagues [120] similarly designed PECs and PEI‐coated liposomes for lipopeptide antigen (LCP‐1) delivery (Figure 9a). Intranasal delivery of three PEC vaccines formulated using distinct PEI coatings (PEI 10 kDa, PEI 25 kDa, and mannose‐PEI 25 kDa) induced antigen‐specific IgG antibody production in BALB/c mice without the need for additional adjuvants. Mannosylated PEI‐based PEC (PEC‐3) showed the highest encapsulation efficiency (99%) and exhibited the strongest immunogenicity (with the highest serum IgG1 titers and ~80% bacterial inhibition), exceeding 25 kDa PEI‐containing PEC (PEC‐2) (70–80%) and PEC‐1 (10 kDa PEI) (~50%) (Figure 9b). However, PEC‐2 demonstrated better stability with a more controlled antigen release profile. Liposomal formulations bearing 25 kDa PEI, either mannosylated (Lip‐3) or nonmannosylated (Lip‐2), elicited higher opsonic antibody titers compared to PECs.

The solid lipid network of SLNs provides numerous advantages over conventional lipid carriers, including a controlled release profile and improved stability [121]. By combining lPEI with the controlled release properties and receptor‐targeting capability of solid lipid components (SLCs), limitations associated with conventional siRNA vectors can be overcome due to sustained release effects [122]. Introducing folate to the surface of PEI‐SLCs improved siRNA delivery efficiency in PC3 cells and prevented nonspecific uptake by RWPE1 cells through receptor‐mediated targeting. This formulation was also significantly more stable under physiological and storage conditions than typical PEI formulations. The SLCs in the system and the receptor‐targeted delivery process minimized both acute and delayed toxicity, preserving the proliferation and viability of noncancerous RWPE1 cells more effectively than conventional PEI‐polyplexes.

Bentley group also developed solid lipid–PEI (SLP) hybrid nanoparticles for topical siRNA delivery, producing nanoparticles with a 200 nm spherical shape, +20 mV ζ potential, and stability for 90 days at 4°C [123]. The combination of SLP containing 0.25% 25 kDa PEI enhanced siRNA delivery to deeper layers of the porcine skin. The transfection efficiency of these nanoparticles was high, with >85% internalization in HaCaT cells and almost 100% in NIH/3T3 cells. These SLPs also produced better knockdown efficiency with a 2.06‐ and 1.67‐fold reduced luciferase expression compared to counterparts with lower PEI concentration and Lipofectamine, respectively. These findings clearly demonstrate superior intracellular delivery and therapeutic potential of PEI‐modified solid lipids.

Natural extracellular vesicles (EVs), also known as exosomes, are excellent drug carriers with high biocompatibility, low immunogenicity, and the ability to evade immune surveillance [124]. Zhupanyn et al. [125] reported significant improvements in the physicochemical and biological properties of EVs when modified with PEI for siRNA delivery. EV‐complexed with PEI polyplexes exhibited larger particle sizes (181 ± 11 nm) compared to unmodified EVs (112 ± 15 nm) and exhibited reduced surface charges (+26 ± 2 mV) compared to PEI‐siRNA complexes (+9 ± 3 mV) – an important factor for reducing cytotoxicity. Under TEM and helium ion microscopy, sonicated EV‐modified PEI complexes displayed a spherical shape, distinct from nonsonicated EVs (aggregates) and standard polyplexes (elongated and irregular). Delivery of siRNA with PEI‐EVs increased luciferase knockdown efficacy, compared to unmodified PEI complexes, and remained active after 5 days of storage. Confocal imaging confirmed the successful co‐localization of siRNA with EVs and showed improved cellular uptake. Enhanced survivin knockdown and significantly reduced tumor cell proliferation were also observed in vitro, as well as significant inhibition of PC3 prostate carcinoma growth in athymic nude mice.

Wallen et al. [126] modified colostrum‐derived exosomes with PEI to facilitate delivery of pDNA and siRNA, mimicking viral gene expression and enabling antiviral candidates to be evaluated under less stringent biosafety measures. This exosome‐based delivery system effectively expressed SARS‐CoV‐2 proteins, including spike glycoproteins, nucleocapsids, and replicases, in vivo and in vitro. In vitro, siRNAs were screened and showed efficient knockdown of viral proteins by 80–95%, while delivery of spike protein and nucleocapsid to mice induced significant antibody production against these antigens for up to 45 days.

### 4.4 Polyethyleneimine‐Functionalized Inorganic Nanomaterials

Several inorganic nanomaterials have been functionalized with PEI to improve their properties, including quantum dots (QDs), CDs, HNTs, gold nanoparticles (AuNPs), mesoporous silica, and magnetic nanoparticles (MNPs). These materials, discussed below in detail, offer unique advantages in biomedical applications due to their inherent structural and chemical characteristics as well as their responsiveness to different stimuli. For example, mesoporous silica can be easily functionalized and capped to enable the loading of therapeutic cargo and controlled release, AuNPs are beneficial due to their photothermal properties, and CDs help bioimaging because of their strong photoluminescence. Attaching PEI to these materials confers positive surface charges that facilitate cellular uptake and endosomal escape. Subsequently, PEI‐decoration protects nucleic acids and enhances drug delivery by stabilizing, protecting, and allowing controlled release.

#### 4.4.1 Polyethyleneimine‐Modified QDs

QDs are nanoscale semiconductor particles that enhance targeted delivery with traceable drug distribution in real‐time by harnessing the fluorescent characteristic of these molecules [127, 128]. Sun et al. [129] used silicon quantum dots (SiQDs) functionalized with PEI and encapsulated in cell membranes of red blood cells to fabricate a biomimetic gene delivery system. Hydrophilic SiQDs were produced through a one‐pot hydrothermal reaction between PEI and 3‐aminopropyltrimethoxysilane, while encapsulation in cell membranes was achieved through extrusion. The DNA‐loaded biomimetic delivery system was more stable with enhanced cellular uptake compared to uncoated SiQDs. Transfection efficiency of 25 kDa PEI‐containing SiQDs was high in 293T cells (77%) and HeLa cells (47%), exceeding bare SiQDs and those containing 10 kDa PEI. Encapsulated SiQD also showed minimal cytotoxicity compared to uncoated ones, performing as porous, safe, and traceable biomimetic gene delivery nanoplatforms.

#### 4.4.2 Polyethyleneimine‐Modified CDs

Nanoscale CDs are carbon‐based nanoparticles with unique optical, chemical, and biocompatible properties [130, 131]. Similar to QDs, CDs enable real‐time tracking of therapeutic agents due to tunable fluorescence. Santos et al. [132] synthesized PEI‐and chitosan‐based CDs via microwave irradiation, hydrothermal synthesis, and a combination of both methods to improve optical and biological properties. The CDs ranged in particle size (1–5 nm) and had a high positive charge density. The synthesis process largely impacted the optical and biological features of the CDs, with PEI‐CDs having the most prolonged photoluminescent emission lifetimes, exhibiting minimal hemolytic activity, yet causing significant cytotoxicity. By contrast, chitosan‐based CDs were highly photoluminescent and displayed high hemolytic activity but produced no cytotoxicity in fibroblasts.

#### 4.4.3 Polyethyleneimine‐Grafted HNTs

**FIGURE 10:**
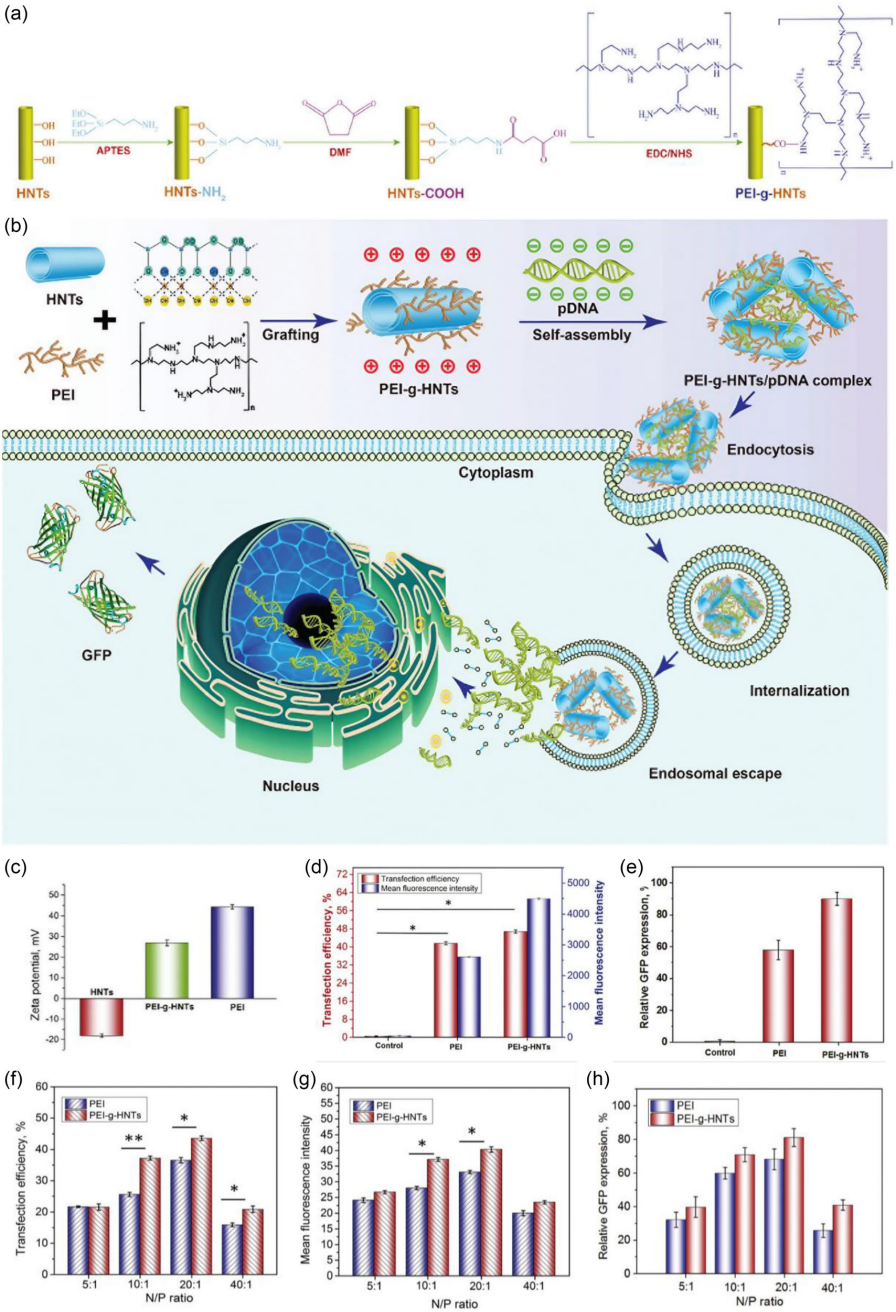
(a) Schematic of PEI‐g‐HNTs synthesis. (b) Schematic showing preparation and assembly of pDNA‐loaded PEI‐g‐HNT complexes and delivery to cells. (c) Zeta potential measurements for HNTs, PEI‐g‐HNTs, and PEI 10 kDa. (d) Evaluation of transfection efficiency, mean fluorescence intensity, and (e) GFP protein expression as determined by western blotting for test groups involving GFP pDNA‐loaded PEI and PEI‐g‐HNT complexes compared to the control. (f) Assessment of transfection efficiency, (g) mean fluorescence intensity, and (h) GFP protein expression obtained from western blotting of GFP pDNA‐loaded PEI and PEI‐g‐HNT complexes at varying N/P ratios. Statistical significance is indicated by *p < 0.05 and **p < 0.01. Adapted with permission [137]. Copyright 2017, Elsevier.

HNTs are naturally occurring aluminosilicate clay minerals characterized by a tubular nanostructure and ranging in diameter from 50 to 200 nm [133, 134]. The hollow lumens and chemically active surfaces of HNTs make them modifiable for more controlled drug and gene delivery [135, 136], while abundance, cost‐effectiveness, and environmental friendliness add to their appeal for theranostic and tissue engineering applications [134]. Long et al. [137] grafted HNTs with PEI as a nonviral gene delivery vector by shortening raw HNTs (365 nm) to ~200 nm, improving endocytosis efficiency (Figure 10a,b). PEI‐grafted HNTs had less cytotoxicity, stronger DNA binding at N:P ratios of 5:1 to 40:1, and reduced ζ potential compared to PEI. The transfection efficiency of PEI‐HNTs was higher in HeLa cells (44%) than PEI alone, and PEI‐HNTs produced more GFP protein than PEI.

#### 4.4.4 Polyethyleneimine‐Functionalized AuNPs

AuNPs possess intriguing properties for drug and gene delivery, especially surface plasmon resonance for optical imaging and photothermal therapy for efficient synergistic use with therapeutic agents [138, 139]. AuNPs are also biocompatible and chemically stable, able to cross biological barriers, making them suitable for targeted delivery with minimal side effects [140, 141]. Lee et al. [142] designed PEI‐functionalized catechol‐coated AuNPs (15–50 nm) for enhanced siRNA delivery via electrostatic interactions and demonstrated that *a* > 2.5:1 weight ratio ensured optimal complex formation. For example, a 5:1 weight ratio enabled the formation of well‐dispersed complexes with no agglomeration present, as observed using TEM. Plasmon resonance confirmed the stability of AuNPs after being complexed with siRNA, while confocal microscopy showed that smaller (15.3 nm) and less charged (+5 mV) complexes exhibited stronger green fluorescence signals compared to larger and more charged counterparts. Gene silencing efficiency in GFP‐expressing MDA‐MB435 cells depended on nanoparticle size and charge, with optimized PEI‐AuNP/siRNA complexes reducing GFP expression to 72%, compared to the conventional PEI system. PEI‐AuNPs also produced less cytotoxicity compared to PEI/siRNA complexes, possibly because no free PEI was present.

#### 4.4.5 Polyethyleneimine‐Functionalized Mesoporous Silica Nanoparticles (MSNs)

MSNs, characterized by large surface areas and tunable pore sizes, are suitable for encapsulating and shielding therapeutic cargo [143, 144, 145, 146]. The surfaces of MSNs are easily modifiable, providing targeted delivery, controlled release, enhanced biocompatibility, and exceptional stability, which contribute to the versatility of MSNs as carriers of drugs, genes, or other biomolecules [147]. These nanoparticles can also integrate diverse stimuli‐responsive mechanisms that further contribute to the potential of MSNs as precise and effective therapeutic delivery platforms [148, 149].

**FIGURE 11:**
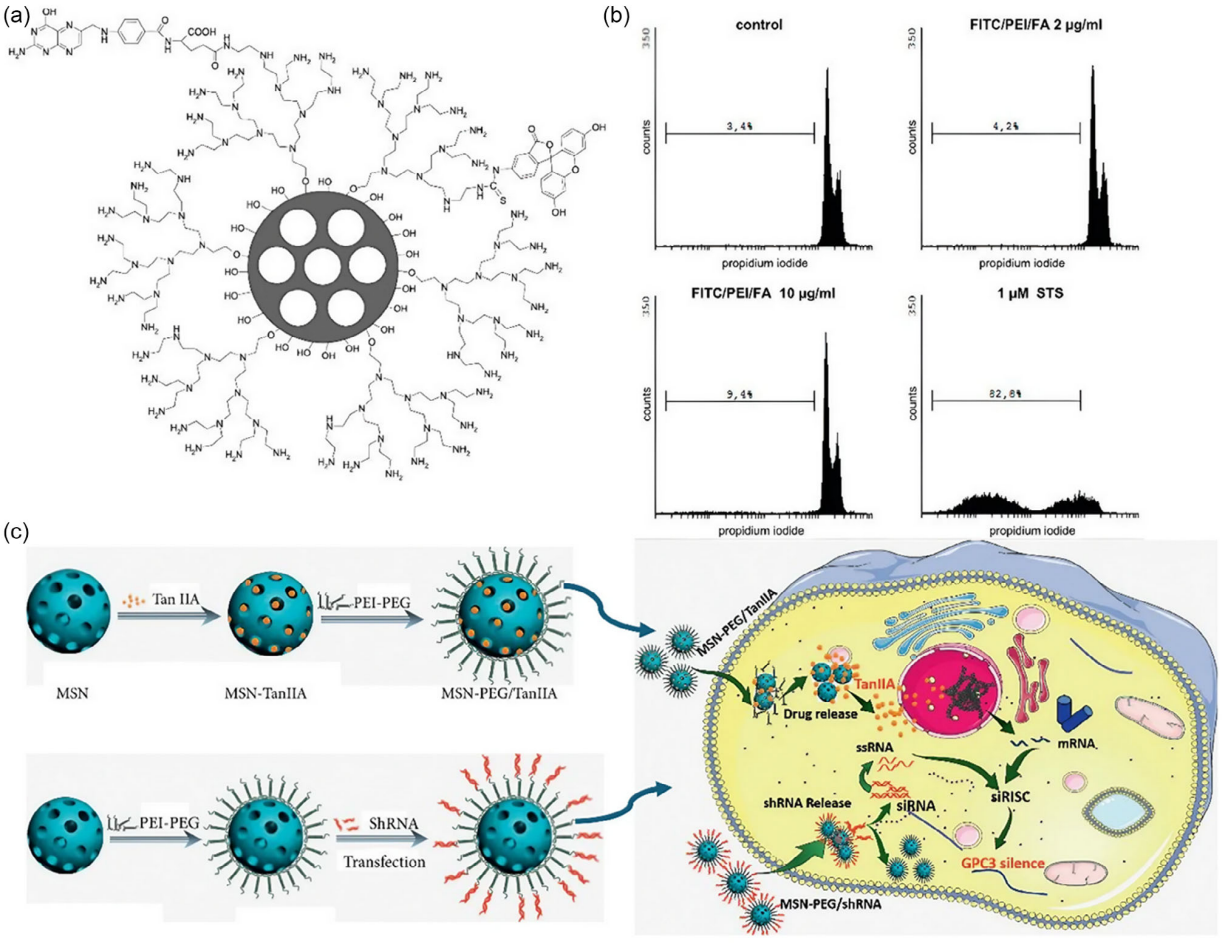
(a) Structure of PEI‐hybrid MSNs. (b) The relative fluorescence intensity of propidium iodide per cell for PEI and FITC/PEI/FA‐hybrid MSNs, negative control, and positive control with 1 μM staurosporine (STS). Adapted with permission [150]. Copyright 2009, American Chemical Society. (c) Schematic of the fabrication and delivery of MSN‐PEG nanoparticles. MSN‐TanIIA‐PEG and MSN‐PEG/GPC3‐shRNA nanocomplexes accumulate in tumors via the EPR effect, facilitating cellular uptake and release of TanIIA into the nucleus and GPC3‐shRNA into the cytoplasm for gene silencing via siRISC. Adapted from [151] under the terms of the CC BY license. Copyright 2021, Zhu et al.

Rosenholm et al. [150] explored the efficiency of PEI‐silica particles for targeted delivery to HeLa cervical cancer cells with MSNs that had an average particle diameter of 400 nm and a high specific surface area. HPEI was functionalized onto the MSN surface via aziridine polymerization, providing sufficient amine groups for further conjugation with folic acid via carbodiimide linkage (Figure 11a). Subsequently, fluorescently labeled, biocompatible nanoparticles were constructed to target cancer cells expressing high levels of folate receptors. Functionalization with PEI decreased pore volume, but the isoelectric point was increased from 7.0 to 9.6, indicating a higher density of amino groups covering the MSN surface. The functionalized folic acid‐modified silica particles were internalized by HeLa cells up to six times more efficiently than embryonic kidney epithelial 293 cells under identical conditions, revealing the selectivity of the fabricated nanoplatform. The nanoparticles were also efficiently endocytosed by HeLa cancer cells via electrostatic attraction and folate receptor targeting, with 52% of cells showing internalization after 24 h. Competition with free folate greatly reduced nanoparticle uptake, confirming a receptor‐based uptake mechanism (Figure 11b). No significant cytotoxicity was induced by PEI‐MSNs as no differences in cell death were observed over 24 h compared to the positive control (Figure 11c).

Another study by Zhu et al. [151] explored the co‐delivery of tanshinone IIA and GPC3‐shRNA to hepatocellular carcinoma using PEG‐PEI‐coated MSNs. The PEI layer on MSN surfaces allowed effective binding to GPC3‐shRNA plasmids and efficient release while protecting them from RNase degradation (Figure 11d). Transfection of HepG2 cells with GPC3‐shRNA‐loaded MSNs achieved 34% ± 1% efficiency, comparable to the 32% ± 1% transfection efficiency of Lipofectamine 2000, and effectively downregulated GPC3 mRNA levels and protein expression. The coated MSNs achieved a sustained drug release of 98% over 24 h with a slower initial release (60% in 6 h) compared to uncoated ones (72%), providing evidence of a tailored release profile. This modified nanoplatform achieved the lowest IC_50_ (7 μg/mL) against HepG2 and the highest inhibition of cell invasion (72% ± 23%), demonstrating superior efficacy for suppressing cancer cell growth and invasiveness.

#### 4.4.6 Polyethyleneimine‐Modified MNPs

Nanoparticles with superparamagnetic properties are gaining interest as drug and gene delivery platforms due to their ability to be precisely controlled and guided by external magnetic fields [152, 153]. MNPs can be easily functionalized with therapeutic agents, polymers, or targeting ligands for improved stability, biocompatibility, and specificity [154, 155]. In addition to drug and gene delivery applications, MNPs can potentially treat cancer through ferroptosis – programmed cell death induced by iron‐dependent lipid peroxidation [156, 157].

Zhao et al. [158] investigated the potential of PEI‐modified MNPs (PEI‐MNPs) as nonviral gene nanovectors and showed that PEI‐MNPs/DNA complexes could be efficiently formed, producing stable transfections with reduced cytotoxicity. PEI‐MNPs exhibited a hydrodynamic diameter of 168 ± 3 nm and a positive ζ potential of +48 ± 1 mV, which decreased upon DNA binding. DNA formed condensed, rod‐shaped structures when MNPs were arranged at low MNP:DNA ratios, while higher DNA concentrations formed “island‐shaped” structures. These complexes also exhibited enhanced transfection potential, prevented DNA deterioration by enzymatic degradation, and exceeded conventional lipofection for expressing GFP. These complexes were also biocompatible across multiple MNP concentrations (0.25 to 1 µg for 10^6^ cells) with cell viability >70% and significantly higher than Lipofectamine. These key observations demonstrated how PEI‐MNPs/DNA complexes could be efficiently formed to produce stable transfections with reduced cytotoxicity.

More recently, Li et al. [159] presented a biomimetic PEI‐based nanogel designed for chemoimmunotherapy and equipped with ultrasmall iron oxide nanoparticles for MRI‐guided treatment of breast cancer. The system was camouflaged via incorporation within the cancer cell membrane. The nanogel delivered docetaxel and CD47 siRNA, effectively restoring macrophage function by downregulating CD47, the “don’t eat me” signal, and inducing immunogenic cell death. In vivo, the system exhibited superior tumor inhibition with 72% tumor growth inhibition. Moreover, combining the nanogel with anti‐PD‐L1 antibodies resulted in 90% reduction in tumor metastasis, while demonstrating minimal organ toxicity.

## 5 Applications of Polyethyleneimine in Therapeutic Delivery

PEI has been extensively explored in gene and drug delivery; however, PEI also exhibits remarkable versatility in vaccine development, serving as a delivery system and an adjuvant. These applications are discussed in detail throughout the following sections.

### 5.1 Gene Delivery

The main goals of gene delivery systems include protecting complex nucleic acids from degradation, promoting cellular uptake of therapeutic cargo [160, 161], and allowing endosomal escape [162, 163]. A wide variety of systems are available for nonviral gene delivery, including liposomes, dendrimers, dendritic mesoporous silica, carbon nanotubes, etc. [164]. Each of these delivery systems has its own advantages for delivering genetic material to cells, although several of these systems have limitations that compromise their delivery efficacy. For example, many of these gene delivery systems struggle to promote significant cellular uptake, evade endosomal entrapment, and maintain stability under physiological settings [165, 166]. Some materials, like QDs, also pose biocompatibility or toxicity concerns, particularly when present in high concentrations that potentially induce nonspecific immune responses or harm healthy cells [167]. Due to their unique capacity for effective cellular uptake and gene expression, PEI‐based vectors possess advantages that other nonviral systems lack in gene delivery (Table 3).

**Table 3:** PEI in gene delivery applications.

Nucleic acid type	Target/Application	Cell/Animal model	PEI formulation	Key outcomes	Ref.
CRISPR/Cas9 pDNA	Multiple genes in MSCs	Mesenchymal stem cells (C57BL/6 mice)	PAMAM (G1)‐ 1.8 kDa PEI‐Arg/‐His/‐HFBA derivatives	• Knockout efficiency (54%–90%) is higher than 25 kDa PEI with lower cytotoxicity • Successful gene editing in primary MSCs with improved safety profile	[34]
CRISPR/Cas9 (Cas9 mRNA + sgRNA)	KRAS mutation in lung cancer	Lung cancer cells	1,2‐epoxytetradecane‐ 0.6 kDa PEI micelleplex	• Indel efficiency (60%) with gene editing efficiency (48.5%) and reduced tumor cell migration • Low toxicity with effective KRAS targeting in lung cancer model	[168]
CRISPR/Cas9 plasmid (px458)	GFP gene knockout	MDA‐MB‐231 breast cancer cells	Bovine serum albumin BSA coated with 1.8 kDa PEI NPs	• Plasmid delivery reached 84.1% ± 1.3% and RNP delivery reached 92.6 ± 1.6% compared to DNAfectamine delivered RNP (83.3%) • Nanoparticles were safe up to 200 mg/kg with no mortality over 30 days	[33]
Cas9 + sgRNA (plasmid, RNP, or plasmid + in vitro sgRNA)	GFP gene editing	HEK 293T‐GFP, HeLa, PC‐12, HUVECs	1.8 kDa PEI‐Arg nanoparticles (linked via arginine‐disulfide linker)	• Increased p/CRISPR delivery to model cells by five–tenfold compared to 25 kDa PEI (either plasmid, RNP, and mixed forms) • Safe delivery to brain tissue with improved membrane permeability and nuclear localization	[31]
CRISPR/Cas9 plasmid (PX459)	TLR‐3	HEK293 cells—expressing TLR‐3	PEI‐coated magnetic nanoparticles (~20 nm)	• Efficient HDR and NHEJ events comparable to Lipofectamine • Nontoxic magnetofection	[169]
CRISPR/SaCas9 (pX601 vector)	Slc26a4 locus editing	Neuro2a cells	25 kDa bPEI	• 25 kDa bPEI formed DNA complexes at N/P ≥6, safe ≤15, with 70.5% GFP transfection at N/P 15 • Cas9/sgRNA delivery induced 9.6%–24.4% indels • Dual sgRNAs produced a 195 bp deletion, enabling multiplex genome editing • Efficiency comparable to Lipofectamine 2000	[32]
mRNA (IL‐2)	Deep‐seated tumors	4T1, HeLa, and A549 cells/syngeneic murine breast cancer model	Complexes of fluorinated PEI‐FCP (fluorinated choline phosphate)	• 3.94‐fold increase in tumor targeting efficacy with deep tissue penetration • High tumor inhibition rate (91.9%) • No abnormal blood parameters;	[170]
pDNA (hIL‐12)	L‐type amino acid transporter 1 (LAT‐1)	HepG2 and 4T1 cells/Mammary cancer expressing LAT‐1	L‐DOPA conjugated 25 kDa bPEI	• L‐DOPA‐PEI polyplexes (≤180 nm with reduced charge density) condensed DNA and achieved 2.5× higher transfection with lower toxicity	[171]
pDNA (pVEGF) with ICG	Photothermal gene therapy	HeLa and HUVECs cells	25 kDa bPEI/ICG/pDNA complex	• In HeLa cells, PI/pGFP with NIR maintained >95% viability, whereas PEI/pGFP reduced viability to <70%. • In HUVECs, PI/pVEGF + NIR achieved 100% scratch closure. • PI/pVEGF + NIR reduced bacteria ≈99.6% and healed wounds (94.4%). • Collagen deposition reached 69.1% in PI/pVEGF + NIR‐treated wounds.	[172]
CRISPR‐Cas9 eGFP pDNA	Pax6 gene (eye development)	Early embryos of *Litopenaeus vannamei*	mPEG‐PEI‐Carbon nanotubes	• PEI‐c‐SWNTs loaded pDNA, increased size, and gained positive zeta potential, transfecting embryos at N/P 10 • PEI‐c‐SWNTs showed low toxicity, cleavage‐stage embryos were most sensitive and survival remained above 40% at high concentrations • CRISPR/Cas9 editing of LvEy and LvToy caused mutations, reduced gene expression, induced eye malformations, and affected embryo survival, with nanoparticle delivery safer than microinjection	[173]
siRNA (Survivin), miR‐155, miR‐1246	Survivin mRNA in prostate carcinoma	Saos‐2 and PC3 cells PC3 prostate carcinoma xenografts	Extracellular vesicles (ECV)‐modified PEI/siRNA complexes	• ECV‐modified PEI complexes achieved ~80% knockdown, ~2.5–6× gene upregulation, >50% cell inhibition, and ~45% tumor growth reduction.	[125]

Abbreviations: HDR, homology‐directed repair; HFBA, heptafluorobutyric anhydride; ICG, indocyanine green; NHEJ, nonhomologous end joining; RNP, ribonucleoproteins; SaCas9, Staphylococcus aureus Cas9; TLR‐3, traffic light reporter.

The first demonstration of PEI as a gene delivery system was in the mid‐1990s when Boussif et al. [3] proved that PEI could condense nucleic acids. Throughout each stage of gene delivery, from complex formation to internalization to endosome escape and nuclear targeting, PEI plays an important role that contributes to its effectiveness as a nonviral vector. A key feature of PEI is an amine‐containing backbone that facilitates strong electrostatic interactions with negatively charged nucleic acids, producing polyplexes that confer the stability of loaded genetic material [30]. However, PEI faces challenges when crossing the plasma membrane due to its large size and hydrophilic nature, so cellular uptake occurs predominantly through endocytosis [174], where PEI bypasses direct membrane penetration by being transported into the cell via cellular vesicles. As detailed in the preceding section 2, endocytosed substances are usually degraded within endosomes, although PEI‐based nanoplatforms are unaffected due to the pH buffering capacity of PEI [7, 175]. Despite the efficiency of PEI‐based polyplexes with endosomal egress, it remains problematic to achieve effective nuclear entry [176].

The transfection efficiency of PEI varies with polymer branching. Nuclear localization of lPEI is more effective than bPEI due to the kinetic instability of lPEI/pDNA complexes under varying salt conditions. As lPEI/pDNA complexes are smaller than bPEI/pDNA complexes, they have a greater tendency to sediment, improving attachment to the cell surface [3]. Another advantage of lPEI polyplexes is a faster disintegration rate with reduced endosomal degradation that further increases transfection efficiency and transgene expression [4, 177]. Gene delivery, and in particular, DNA delivery, must overcome the challenge of reaching the nucleus once released into the cytoplasm. This can occur when cells divide and the nuclear envelope is disassembled [178, 179]; however, plasmids can also be chemically modified to enhance nuclear entry outside of cell division phases. Enhancing the nuclear uptake of plasmid DNA requires coating plasmids with nuclear localization signals or pre‐complexing plasmids with transcription factors, like serum response factor or neurokinin‐3 [180, 181]. Other strategies exploit importin β‐binding domains and glucocorticoid receptor activation with dexamethasone to boost gene expression [180].

Therapeutic use of genetic materials, such as pDNA and small interfering RNA (siRNA), has shown promise for treating various diseases, including cancer, viral infections, and neurological disorders, by selectively targeting and inhibiting specific messenger RNA (mRNA) sequences [182]. The design of reliable nonviral gene delivery systems requires careful consideration of multiple factors to ensure the effective and safe transfer of genetic material [183]. The particle size of the formulation is crucial. For cellular uptake via endocytosis, 50–200 nm particle sizes are preferred as larger particles are unlikely to enter the cells or may be cleared rapidly [184, 185]. Nanoparticle's ζ potential is another important factor, as a balanced cationic charge promotes cellular uptake while minimizing cytotoxicity. The N:P ratio also plays a pivotal role in effectively condensing DNA without increasing polymer cytotoxicity [186]. Ensuring formulation stability is another important criterion for PEI‐based nanocarriers to prevent premature DNA release under physiological conditions. Nonviral vectors should also exhibit tunability to improve biocompatibility and target specificity while minimizing immune reactions [110].

Gou et al. [187] recently developed heparin‐PEI (h‐PEI) nanogels to deliver pDNA encoding vesicular stomatitis virus matrix protein (pVSVMP), which inhibits tumor growth by inducing apoptosis. Heparin was selected for its biocompatibility and targeting potential to enhance the safety and efficacy of the delivery system. The nanogels, formed by conjugating heparin to PEI through biodegradable amide bonds, had an average size of 75 ± 7 nm in hydrated form with a ζ potential of +27 ± 1 mV. In vitro studies showed that h‐PEI nanogels efficiently transfected pVSVMP into C‐26 colon cancer cells, inducing apoptosis and significantly reducing proliferation. The transfection efficiency of the h‐PEI nanogels was comparable to that of 25 kDa and 2 kDa PEIs, but produced significantly less cytotoxicity (IC_50_ = 77 μg/mL) than 25 kDa PEI (<10 μg/mL). The h‐PEI nanogels also exhibited higher biocompatibility than 25 kDa PEI, as hPEI‐nanogels did not cause hemolysis or erythrocyte aggregation. In mice treated with pVSVMP/h‐PEI complexes, the mean tumor weight of abdominal metastases was significantly less (0.93 g) than that of the control (3.28 g), and survival rates were higher. Notably, heparin‐modified PEI nanogels exhibited higher biocompatibility than 25 kDa PEI, and no acute toxicity was observed in response to the modified nanogels even when more than twice the dose of 25 kDa PEI was administered.

**FIGURE 12:**
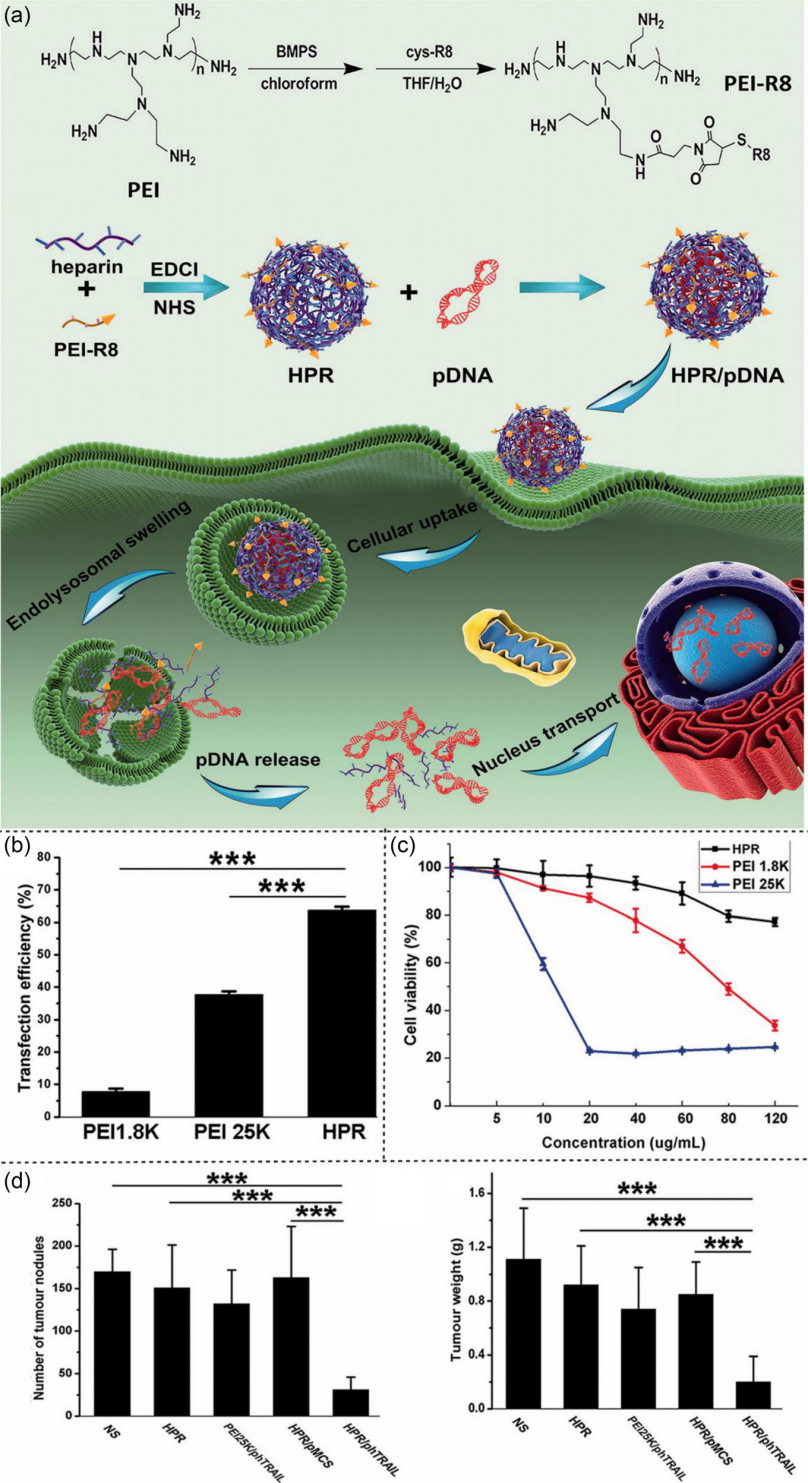
(a) Diagram depicting synthesis of PEI‐R8 along with the formation of HPR/phTRAIL nanoparticles for gene delivery. (b) In vitro transfection efficiency percentage and (c) cytotoxicity of HPR compared to 1.8 kDa PEI, 25 kDa PEI in HCT‐116 cells. (d) Tumor weight and number of nodules across treatment groups: normal saline control (NS), HPR, 25 kDa PEI/phTRAIL, HPR/pMCS, and HPR/phTRAIL. Adapted from [188] under the terms of the CC BY license. Copyright 2018, The Authors. Published by Taylor & Francis.

Song et al. [188] developed a targeted nanogel composed of heparin and cell‐penetrating octa‐arginine peptide (R8)‐modified lPEI (HPR) (Figure 12a). In this system, heparin effectively mitigated PEI cytotoxicity (Figure 12c) by shielding the primary PEI amine groups and decreasing cellular stress and membrane damage typically caused by cationic polymers. No adverse effects were detected by blood chemistry tests or organ histology. The R8 peptide enhanced the cellular uptake and endolysosomal escape of the nanogel, increasing its gene delivery efficiency. The HPR nanogels effectively delivered pDNA encoding human tumor necrosis factor α (TNF‐α)‐related apoptosis ligand (phTRAIL) to HCT‐116 cells, causing significantly higher apoptosis (42%) than 25 kDa PEI/phTRAIL (19%), showing the superiority of HPR over 25 kDa PEI in gene delivery. Overall, HPR exhibited a high transfection efficiency in vitro and promising antitumor activity in vivo by reducing tumor size and nodules in a model of abdominal metastasis (Figure 12b,d). Zhou et al. [189] further investigated the effectiveness of heparin‐PEI‐plasmid nanogels. In their study, nanogels interleukin‐15 were loaded for lung‐targeted gene delivery, aiming at plasmid distribution, tumor metastasis inhibition, immune response, and effects on tumor cell apoptosis and proliferation. Compared to 2 and 25 kDa PEI‐based complexes, the heparinmodified complex delivered the most plasmid into lung metastases of CT26 colon carcinoma and B16‐F10 melanoma. In mice treated with these complexes, natural killer cells were increased in tumors, along with increased levels of TNF‐α and IFN‐γ observed in serum. Consequently, the tumor metastatic index was reduced, and apoptosis was induced, which contributed to inhibited proliferation in lung tumor sites.

### 5.2 Drug Delivery

Controlled and sustained drug delivery systems have been introduced to prolong circulation time and optimize therapeutic effectiveness, especially for low‐molecular‐weight therapies that are rapidly cleared from circulation by the kidneys and taken up by organs of the reticuloendothelial system. The development of synthesis methods and novel bioactive materials has set the stage for biomimetic drug delivery systems. Inspired by natural biological mechanisms to enhance drug delivery and targeting, these biomimetic drug delivery systems support and stabilize the drug while other components or techniques strive to control release, localization, and responsiveness [28, 189].

Polymeric drug delivery systems exist in several forms designed to address specific therapeutic needs, including PECs, porous nanoparticles, nanogels, and emulsions. Several PEI‐based platforms have been developed to enhance the co‐delivery of therapeutic agents in cancer treatment (Table 4). Li et al. [199] designed a delivery system for two hydrophobic anticancer drugs, 10‐hydroxycamptothecin and paclitaxel, that consisted of Pluronic F127 micelles crosslinked with PEI nanogels. In vitro, only 15% of paclitaxel was released in the first 5 h from F127/PEI nanogels compared to 30% from F127 micelles, indicating that PEI modification reduced initial release by 50%. Cytotoxicity assessment of paclitaxel‐loaded F127/PEI nanogels against HepG‐2 hepatic cancer cells produced a lower IC_50_ value (1.2 ± 0.1 μg/mL) than free paclitaxel (29 ± 3 μg/mL). Meanwhile, folate modification enhanced cytotoxicity by reducing the IC_50_ value to 0.40 ± 0.03 μg/mL.

**Table 4:** Summary of PEI‐based co‐delivery nanosystems for cancer therapy.

Study ID/Ref.	Therapeutic cargo	Cancer cells	Carrier system	PEI, MW	Size, nm	Zeta potential (ζ), mV	EE%/DL%	Targeting	Release, pH 7.4/5–6	Key outcomes
Navarro et al. 2012 [190]	DOX siMDR‐1	MCF‐7 (breast, MDR resistant)	DOPE‐PEI conjugate (PEI‐glutaryl‐DOPE)	Branched 1.8 kDa	120–180	+22 ± 5	—	None (passive)	—	• Complexed siRNA at N/P ≥3 and protected 92% from RNase III for 2 hr • In c166 GFP cells reduced fluorescence 2.5–fourfold with 75% silencing at N/P 16 • In resistant MCF‐7 cells reduced P‐gp expression and doubled intracellular DOX • Sequential siMDR‐1 + DOX reduced viability from 95% to 45% at 24 h and to 16% at 2–3 days
			MNPs: DOPE‐PEI core + POPC/Chol/PEG‐PE shell	Branched 1.8 kDa	127 ± 2 to 178 ± 34	+3 ± 1 (PEG shielded)	—	None (stealth PEG)	—	• Achieved gene silencing comparable to Lipofectamine 2000 with about 100% viability versus 50% • In resistant MCF‐7 cells reduced P‐gp expression and doubled intracellular DOX • Sequential siMDR‐1 + DOX reduced viability to 65% at 24 h and to 16% at 2–3 day
Vural et al. 2026 [191]	DOX miRNA‐424‐3p	A549 (lung)	DMSN‐LbL (DMSN → PEI → miRNA → PLL)	Branched 25 kDa	164 ± 7	+28 ± 2.3	DOX EE%: 90% miRNA EE%: 86%	None (passive EPR)	<10%/>90% (lysosomal, 3 h)	• Co‐delivery reduced A549 viability to 49 ± 2% (200 µg/mL, 24 h); 49% late apoptosis; Gal3 protein dropped by ~ 50% (translational repression)
			DMSN‐LbL + anti‐Gal3 Ab (DMSN → PEI → miRNA → PLL → Ab)		171 ± 4	+31.5 ± 2.4	—	Receptor‐mediated (anti‐Gal3 mAb)	—	• Ab functionalization increased late apoptosis to 56.27%, further enhancing Gal3 inhibition and pro‐apoptotic effect
Wang et al. 2026 [192]	DOX Bcl‐2 siRNA	HepG2 (HCC)	FA‐mPEG‐PCL‐PEI triblock amphiphilic micelle@Fe_3_O_4_	Branched 0.8 kDa	~225	~+20 (PBS); ~+10 (+10% FBS)	EE: 84.26% DL: 2.91%	Dual: FA (folate‐Receptor mediated) + magnetic targeting	~40% (85 h) pH 5.7 ~80% (85 h) pH 7.4	• Drug‐loaded micelles showed stronger T2 MRI contrast than free Fe3O4. • Hep‐G2 viability decreased to 37.32% with apoptosis reaching 67.0%. • Migration reduced to 15.00% versus 55.34% control. • Bcl‐2 expression decreased to 55.69% with folate micelles. • ROS levels exceeded free DOX. • Micelles + magnetic targeting achieved 80% inhibition by day 22. • Two of three mice survived beyond 40 days with tumor fluorescence increased with folate and magnetic targeting and tumors were smallest in magnetic micelles group with no necrosis detected in major organs
Liu et al. 2025 [193]	Cisplatin PRMT5‐shRNA	PC9‐NED & A549‐NED (NSCLC, cisplatin‐ resistant NED)	MSN‐PEI/shRNA NPs in HA‐MA hydrogel (UV‐crosslinked; HAase‐responsive; CD44‐targeted)	Branched PEI (MW unspecified)	bare MSN: 216.6 MSN‐PEI: 285.9	bare MSN: −0.33 MSN‐PEI: +46.21	EE: 78.60 ± 0.21% DL: 34.70 ± 0.75%	CD44 receptor targeting via HA	in HAase: ~10% (144 h) in HAase: ~60%–65% (144 h)	• HA‐MA + cisplatin@MSN–PEI/PRMT5‐shRNA hydrogel reduced PC9‐NED and A549‐NED cell viability to 30% and 15% at 11 µM cisplatin, respectively, outperforming cisplatin alone. • PRMT5, MDR1, and BCRP gene expression decreased by 50%–60%. • Late apoptosis increased from 6 to 8% (control) to >50% in PC9‐NED and 43% in A549‐NED cells. • Normal BEAS‐2B cells maintained 80%–90% viability, showing low toxicity.
Meng et al. 2022 [194]	PTX siPD‐L1 or CDN (STING agonist)	CT26 (colon), B16F10 (melanoma), 4T1 (breast)—murine	PEI‐LCA conjugate (2E’) Amphiphilic self‐ assembling NP	Branched PEI (unspecified MW)	264.4	+44.6 ± 0.3 (2E’/PTX) +28.9 ± 1.0 (+siPD‐L1)	PTX EE: 81.9% DL: 23.4 ± 6.3 wt%	None (Direct intratumoral injection)	32% PTX in 72 h	• All PEI‐treated mice died from acute toxicity, while no deaths occurred in the PEI–lithocholic acid group during the study period • Single intratumoral injection achieved 86% complete tumor regression and markedly reduced lung metastases • CD8 T cell infiltration increased over twofold, PD‐L1 decreased, and rechallenge resulted in complete tumor rejection
Tang et al. 2025 [195]	DOX (encapsulated) siPD‐L1	CT26 (colon)	β‐CD‐OEI/mPEG‐β‐CD/ Ad‐PCL‐Ad supramolecular micelles (I_1_G_5_A_1_; 1:5:1)	0.6 kDa OEI; 7 arms on β‐CD	Unloaded: 142–156 DOX‐loaded: 173–182	+3.6 ± 0.5 (bare); nearly neutral w/siRNA	DOX EE: 77.8 ± 1.3% DL: 3.9 ± 0.2%	None (passive EPR); PEG stealth; modular design	pH 7.4: ~30% pH 5.1: ~50% (100 h)	• Dox uptake peaked at 2 h • IC50 was 0.25–0.5 mg per mL • No toxicity up to 1 mg per mL • PD L1 expression markedly decreased after siRNA delivery
Hosseini et al. 2026 [196]	DOX + EGFP plasmid (reporter gene)	HepG2 (HCC)	GO–AuNP–PEI–PEG– DOX–EGFP nanoplex	Branched PEI 25 kDa	160 ± 4	−2.05	EE: 96.9 ± 1% DL: 33.9 ± 0.5%	Passive (EPR)	~2× faster at pH 5.6 versus pH 7.4 (120 h)	• GO‐Au‐PEI‐PEG‐DOX at 12.5–25 µg/mL reduced HepG2 viability more than free DOX, while preserving higher L929 viability. • At 72 h, cancer cell death exceeded normal cells, with significantly higher transfection than PEI control.
Hailing et al. 2020 [197]	DOX	MHCC‐97L, Hep3B (liver HCC)	Carbon dot passivated with bPEI & DOX (CD–PEI–DOX)	Branched 25 kDa	2–8	+11.5	EE: 35.88%	None (passive EPR)	pH 7.4: 7% pH 5.0: 72% (72 h)	• CD‐PEI‐DOX killed MHCC‐97L and Hep3B cells selectively; 50% survival in MHCC‐97L, 70% in L02 at 10 μg/mL. • Cellular uptake was 4.7× higher than DOX • Apoptosis in MHCC‐97L increased to 20.95% versus 13.06% for free DOX. • In vivo, reduced tumor growth, maintained body weight, high tumor accumulation, low systemic toxicity, inhibited Ki67, increased cleaved caspase‐3.
Li et al., 2016 [198]	DOX VEGF shRNA	MCF‐7 (breast), HeLa (cervical)	MSN‐DOX‐PEI‐FA; MCM‐41 MSN + PEI via APTES	10 kDa	217	~+32 (N/P 10)	DL: 12.3% (DOX: M‐MSN = 0.4:1)	Folate receptor‐mediated (FA)	pH 7.4: ~60% pH 5.0: ~35% (72 h)	• HeLa viability decreased to 27% with DOX shRNA nanocomplex + magnetic field • DOX uptake increased 1.7‐fold with folate targeting • VEGF silencing reached 90.4% • VEGF protein decreased from 1640 to 578 pg per mL

Moreover, Ahsan group [200] explored the effect of PEI on the nasal absorption of the negatively charged drug, enoxaparin. PEIs of various molecular weights were combined with enoxaparin to form complexes that reduced the negative charge of enoxaparin, associated with sulfate and carboxylate anionic groups, and enhanced its nasal absorption. This neutralization process weakened the Coulombic repulsion of the drug with the negatively charged cell membrane, enhancing drug uptake. Similar to PEI‐enhanced DNA transfection, PEI could bind to negatively charged molecules on the cell surface, triggering endocytosis‐mediated drug uptake. Findings showed that 1000 kDa PEI was the most effective formulation, followed by 750 kDa PEI and 25 kDa PEI. In particular, when 0.25% 1000 kDa PEI was combined with enoxaparin, the bioavailability of enoxaparin was fourfold higher than the free drug.

Tiyaboonchai et al. [201] developed a PEI‐dextran sulfate nanoplatform for insulin delivery with enhanced stability, prolonged therapeutic effects, and administration through nonparenteral routes. Insulin was incorporated into this system via self‐assembly by mixing in Tris buffer with zinc sulfate to reduce particle size and improve stability. To achieve stability of these nanoparticles as well as the insulin secondary structure, high PEI:dextran ratios (up to 3:1) and zinc sulfate concentrations (15–25 μM) were used, which influenced α‐helix to β‐sheet transitions. This resulted in spherical nanoparticles with a 250 nm diameter and *a* +30 mV ζ potential, and had high insulin entrapment efficiencies (up to 90%) with no evident insulin degradation. Despite the rapid in vitro release of these nanoparticles, they exhibited sustained hypoglycemic effects in vivo [201].

Another instance of protein transduction was when Kitazoe et al. [202] delivered biotinylated proteins (e.g., eGFP and RNAse A) by PEI‐streptavidin, PEI‐avidin, and PEI‐protein G systems. Indirect PEI‐cationization simplified interactions with biotinylated proteins via noncovalent bonding while preserving protein functionality. Biotinylated proteins provided cleavable disulfide bonds that could help liberate loaded proteins inside the cell. Proteins transduced by PEI‐cationized carriers also retained functionality; biotinylated GFP was localized to nuclei, while biotinylated RNase A derivatives delivered by PEI‐streptavidin induced cytotoxicity by degrading intracellular RNA. Similarly, anti‐S100C antibodies delivered via PEI‐protein G exhibited precise intracellular targeting in human fibroblasts.

Neurodegenerative diseases remain challenging to diagnose, manage, and prevent due to the selective permeability of the blood‐brain barrier (BBB) [203], which limits the therapeutic potential of nearly all macromolecules and over 98% of small molecules [110, 204]. This challenge requires extensive research into how effective delivery systems can be designed to facilitate transport across the BBB. Kabanov et al. [205] developed cationic nanogels (~100 nm) composed of cross‐linked PEG and PEI chains as brain‐directed delivery systems for antisense oligonucleotides. When these nanogels were modified with transferrin and insulin, they crossed the BBB more efficiently. In other studies, Moore and coworkers [206] developed PEI‐perphenazine nanoparticles that modulate amyloid aggregation in Alzheimer's disease, transforming toxic Aβ40 oligomers into nontoxic prefibrillar forms, while Otzen and colleagues [207] constructed PEI‐coated albumin nanoparticles targeting α‐synuclein, a protein implicated in the pathogenesis of Parkinson's disease. Richter group [208] developed an intracerebroventricular infusion of PEI‐complexed siRNA targeting α‐synuclein mRNA, which reduced mRNA expression by 65% and α‐synuclein levels by 50% in the striatum of a mouse model of Parkinson's disease. The positive charges of PEI facilitated transport across the BBB and formed strong interactions with α‐synuclein, reducing harmful oligomer formation. Importantly, this delivery system exhibited extensive CNS distribution with no signs of toxicity or immune response.

### 5.3 Vaccine Adjuvants and Delivery

Polyethyleneimine has shown remarkable versatility in vaccine development, serving not only as a vaccine delivery system but also as an adjuvant [7]. Nearly all modern vaccines contain adjuvants that boost immune responses and improve vaccine effectiveness [209, 210], which require stringent safety and efficacy testing before being commercialized [209]. PEI is a promising adjuvant and immunostimulatory agent that enhances antigen uptake and immune activation; however, the safety profile of PEI is incomplete and requires further research to establish the potential of this polymer in vaccine development.

Injectable vaccines are effective for systemic immunity, although they often fail to protect against pathogens that enter through mucosal surfaces, such as HIV‐1, influenza A, and HSV‐2 [211, 212]. Mucosal entry points are the first point of contact between these pathogens and the immune system, making them primary targets for vaccine administration. Mucosal vaccines stimulate local mucosal immunity (primarily through IgA antibodies) and systemic immunity (through IgG), which is particularly effective against pathogens entering the body via mucosal contact, including the respiratory and gastrointestinal tracts [213, 214]. Mucosal vaccines are also noninvasive, increasing patient comfort and minimizing bloodborne transmission risk associated with mass vaccination programs [215, 216]. In 2012, Wegmann et al. [217] investigated PEI as a mucosal adjuvant by immunizing mice intranasally with a recombinant HIV‐1 gp140 glycoprotein complexed with different PEI forms or the cholera toxin B subunit (CTB), a well‐studied mucosal adjuvant. Antigen‐PEI complexes were prepared with PEI of different molecular weights (25 kDa, 40 kDa, 160 kDa, and 750 kDa) by mixing antigens with pre‐diluted PEI solutions and letting them sit for at least 2 h before immunization. Serum IgG titers were ~6‐ and 100‐fold higher than CTB and bare antigen, respectively, while IgA titers in response to 25 kDa and 750 kDa bPEI groups were tenfold higher than the CTB treatment group.

Dong et al. [218] modified Rehydragel with PEI to enhance antigen delivery and improve antigen (ovalbumin) cross‐presentation by DCs. These nanoparticles were composed of high‐purity aluminum hydroxide and heparanase nanoparticles used in clinical applications. The PEI in this formulation enhanced the maturation and activation of tumor‐specific T cells by aiding antigen delivery and cross‐presentation in DCs. The Rehydragel‐PEI complex significantly increased interleukin 12 (IL‐12) secretion by DCs, increased expression of CD80 and CD86 molecules, induced tumor‐specific T‐cell responses, and inhibited tumor growth in mice.

Dai et al. [118] developed a liposome‐based intranasal mucosal vaccine using PEI to deliver a lipopeptide antigen derived from GAS M protein. Four types of liposomes were investigated: neutral liposomes consisting of dipalmitoylphosphatidylcholine and cholesterol; nonlipidated PEI liposomes incorporating branched 600 kDa bPEI; liposomes with lipidated bPEI (conjugated to palmitic acid or dioleoylphosphatidylethanolamine); and the positive control (experimental liposomes) incorporated the cationic lipid, dimethyldioctadecylammonium bromide. Immune responses were assessed in response to the vaccine constructs and showed that the nonlipidated PEI liposome was the most effective, inducing the highest IgA and IgG antibody titers and displaying significantly more robust opsonic activity (60–75%) against five clinical GAS isolates compared to other formulations and the negative control. Notably, PEI‐liposomes performed well as self‐adjuvant platforms for delivering GAS peptide antigens, providing evidence of PEI as a promising tool for protection against GAS infection.

Sun et al. [204] also synthesized PEI triethyleneglycol (TEG) complexes using 2 kDa PEI and 0.6 kDa PEI. They also prepared a mannosylated version of the PEI–TEG complex to assess cellular uptake, cytotoxicity, and delivery efficiency. At a 3:1 mass ratio, the complexes protected DNA from being degraded by DNase. The transfection efficiency of mannosylated PEI‐TEG in DC2.4 cells was threefold higher than the nonmannosylated counterpart (*p* < 0.01). A significant improvement in cell viability (>80%) was observed with mannosylated‐PEI–TEG derivatives when N:P ratios were increased up to 50:1. These complexes also induced maturation markers (CD40, CD80, and CD86) in bone marrow‐derived DCs, confirming DC activation while preserving cell viability. Researchers from Sun's group also reported the effective use of mannosylated‐PEI to increase the activity of a DNA vaccine encoding an HIV gag fragment [219]. Since mannose receptors are highly expressed on APCs, mannosylation was proposed to boost transfection efficiency in DCs. These mannosylated complexes increased gene expression in mice by 600‐ and fourfold compared with naked DNA or 25 kDa PEI alone, respectively. Even at a lower dose of DNA, the mannosylated complexes induced higher T‐cell responses, cytokine production, and memory CD8+ T‐cell activation than naked DNA.

Hollow mesoporous silica nanoparticles (HMSNs) have also emerged as exciting formulations for antitumor vaccines as they exhibit adjuvant‐like properties and induce Th1‐mediated immunity against tumors [220, 221]. However, the advantages of HMSNs are limited by disadvantages like low CD8+ T cell activation and low IgG titers. Negatively charged surfaces of HMSNs also impede antigen uptake by DCs, leading to compromised efficacy. To address these limitations, HMSNs with extra‐large mesopores were developed for antigen delivery in cancer immunotherapy. The hollow structure was fabricated by removing multi‐core iron oxide assemblies, providing high protein loading capacity. When these MSNs were coated with PEI, they were able to load antigens and serve as an immune adjuvant, stimulating DC activation. In vitro, PEI‐coated MSNs enhanced DC uptake, antigen retention, and expression of key activation markers (CD86 and MHC‐II). Similarly, PEI‐coated MSNs significantly increased antigen‐specific cytotoxic T lymphocytes, suppressed tumor growth, and improved survival rates in a mouse model of melanoma.

Furthermore, Liu et al. [222] synthesized PEI‐modified thin‐shell hollow MSNs (THMSNs) and loaded them with the tyrosinase‐related protein 2 (Trp2) antigen peptide to evaluate improvements in loading efficiency, release profile, and cellular uptake. HMSNs modified with PEI exhibited moderate loading (9%) and high encapsulation efficiency (95%) compared to unmodified HMSNs, which had a loading efficiency of 2% and encapsulation efficiency of 22%. PEI‐modified HMSNs displayed a controlled release profile, with 12% peptide release in the first 30 min and 76% over 72 h, while untreated HMSNs showed burst release by releasing 31% in the first 30 min and 94% within 4 h. Modified HMSNs were also taken up by cells more efficiently (~90%) than untreated HMSNs (70%) and exhibited improved adjuvant ability by significantly stimulating DC maturation, with a higher percentage of CD86+ and CD80+ positive DCs (71 ± 1%) compared to HMSNs (54 ± 2%) and increased levels of proinflammatory cytokines IL‐1β, TNF‐α, and IL‐6. On day 20, Trp2 antigen‐loaded THMSNs induced effective cellular immunity and significantly inhibited tumor growth, reducing tumor weight by >80% to <0.5 g, compared to ~4 g in the control group.

## 6 Clinical Trials

The use of RNA‐based therapeutics has increased in recent years, particularly the use of mRNA vaccines that gained increased interest since the remarkable success of mRNA vaccines against COVID‐19. Several trials have explored JetPEI, a 22 kDa lPEI, as a gene delivery product. JetPEI formulations, like BO‐112, CYL‐02, and IFx‐Hu2.0, are being developed for innovative gene and vaccine therapies (Table 5). For example, CYL‐02, conjugated with JetPEI and combined with gemcitabine, was tested in a 2015 phase I trial (NCT01274455) for pancreatic adenocarcinomas [223]. CYL‐02 was well tolerated with no dose‐limiting toxicities, expressed therapeutic RNA in injected tumors with minimal systemic exposure, and inhibited tumor progression effectively. BO‐112, another JetPEI‐based formulation composed of a dsRNA (poly I:C) that activates immune pathways such as TLR3 and RIG‐I, inducing immunogenic cell death, is also being explored. Trials are underway for BO‐112, combined with immune checkpoint inhibitors, in soft tissue sarcomas (NCT04420975), malignant melanoma (NCT04570332), and refractory nonsmall cell lung cancers (NCT05265650).

**Table 5:** Clinical studies of PEI‐based treatments.

Therapeutic agent	NCT	Condition/s	Phase	PEI Mw	PEI formulations	Combination therapy	Dose regimen	No. of subjects	Outcome	Start date	End date
DermaVir	NCT00712530	HIV	I	Mannosylated‐22 kDa lPEI	pDNA/PEI	N/A	Single dose of (0.1, 0.4, 0.8) mg pDNA	9	• lPEI was safe and well‐tolerated at all doses. • Significant increase in HIV‐specific memory T‐cell responses.	2005	2006
	NCT00270205		II			N/A	Cohort 1: 0.1, 0.4 mg pDNA at weeks 1/7/13	25	• The intermediate dose of DermaVir achieved the highest PHPC increase and viral load reduction but showed low activity and significant T‐cell responses only at week 17.	2006	2010
							Cohort 2: 0.4 mg pDNA in week 1/7/13				
							Cohort 3: 0.8 mg pDNA in week 0/1/6/7/12/13				
	NCT00711230		I/II			N/A	0.2, 0.4, 0.8 mg pDNA at weeks 0/6/12/18	36	• The 0.4 mg dose had higher immunogenicity and more viral load reduction. • Significant increase in HIV‐specific memory T‐cell responses.	2008	2015
GEN‐1 (formerly EGEN‐001, now IMNN‐001)	NCT00137865	Recurrent ovarian cancer	I	1.8 kDa bPEI	pDNA/PEG‐PEI‐Cholesterol		0.6, 3, 12, or 24 mg/m^2^ at weeks 1/2/3/4	13	• bPEI was safe and well‐tolerated. • Measurable levels of IL‐12 plasmid and treatment‐related increases in IFN‐g levels in PF samples	2005	2006 (terminated)
	NCT03393884	Recurrent ovarian epithelial cancer, fallopian tube cancer, or primary peritoneal cancer	I/II			Paclitaxel/ Carboplatin	Paclitaxel 175 mg/m^2^ + Carboplatin AUC 6 IV on Day 1 (q3w, 6 cycles), whereas IMNN‐001 dose was administered 100 mg/m^2^ intraperitoneally (17 doses).	110	N/A	2018	Ongoing (2025)
	NCT05739981		II			Bevacizumab/Paclitaxel/ Carboplatin	IMNN‐001 (80 mg/m^2^ IP) is given weekly during adjuvant therapy, then every 21 days with Bevacizumab for up to 18 cycles.	Estimated (50)	N/A	2023	Ongoing (2028)
BC‐819/PEI	NCT01878188	Bladder cancer	I	25 kDa bPEI	pDNA/PEI	Bacille Calmette–Guérin (BCG)	BC‐819 was administered intravesically in combination with BCG using three schedules: alternating, sequential, and twice weekly for 6–12 weeks.	38	• The therapy was well‐tolerated, with five mild adverse events related to BC‐819. • Following 24 months, recurrence and progression rates were 46% and 24%, respectively.	2013	2017
IFx‐Hu2.0	NCT04160065	advanced nonmelanoma skin cancer	I	JetPEI	pDNA/PEI	N/A	0.1 mg pDNA/PEI—monotherapy—up to 3 time points.	20	• IFx‐Hu2.0 was safe and well‐tolerated, with no severe adverse events.	2020	2024
CYL‐02	NCT01274455	Advanced pancreatic adenocarcinoma	I		pDNA/PEI	Gemcitabine	CYL‐02 was tested at four escalation doses between 125 and 1000 µg alongside IV gemcitabine (1000 mg/m^2^).	22	• CYL‐02 was expressed in the injected tumors and, to some extent, was distributed within the bloodstream.	2010	2013
	NCT02806687		II				Cohort 1: 1 mg CYL‐02 + gemcitabine (1000 mg/m^2^); Cohort 2: gemcitabine (1000 mg/m^2^).	68	N/A	2017	2022
BO‐112	NCT04570332	Unresectable malignant melanoma	II		dsRNA (poly I:C)/ PEI	Pembrolizumab	BO‐112 is administered intratumorally at 1–2 mg per session to 1–8 lesions weekly for 7 weeks, then every 3 weeks, with pembrolizumab given intravenously every 3 weeks.	42	N/A	2020	2024
	NCT04420975	Resectable soft tissue sarcoma	I			Nivolumab	BO‐112 is administered intratumorally at 1 mg on days 1, 8, and 15, with nivolumab intravenously at 240 mg on days 8 and 22, followed by 5 fractions of neoadjuvant radiotherapy between days 8 and 12.	14	N/A	2020	Ongoing (2026)
	NCT05265650	Metastatic refractory nonsmall cell lung carcinoma	Ib/II			Nivolumab plus stereotactic ablative radiotherapy (SABR)	BO‐112 is injected intratumorally at 1–2 mg weekly in cycle 1, then every 2 weeks. Nivolumab is given at 240 mg IV every 2 weeks, starting week 7 (cohort A) or week 5 (cohort B). SABR starts in week 3.	30	N/A	2022	2024
MK‐4621	NCT03065023	Recurrent Solid Tumors	I/II		dsRNA/PEI	N/A	MK‐4621 was administered intratumorally in four dose levels (0.2–0.8 mg) twice weekly over a 4‐week cycle.	15	• MK‐4621 monotherapy and combination therapy with pembrolizumab demonstrated tolerable safety, modest antitumor activity, and activation of the RIG‐I pathway but provided no meaningful clinical benefit at the tested doses.	2017	2018 (terminated)
	NCT03739138		I/Ib			Pembrolizumab	MK‐4621 was administered intratumorally at doses of 0.2–0.8 mg weekly in 3‐week cycles for up to 6 cycles, alongside 200 mg pembrolizumab every 3 weeks for up to 35 cycles.	30		2018	2021 (terminated)
DNA vaccine	NCT04049864	Relapsed neuroblastoma	Early phase I	20 kDa bPEI	pDNA/PEI	N/A	Cohort 1: 20 μg pDNA/cm^3^ tumor. Cohort 2: 40 μg pDNA/cm^3^ tumor. Administered intratumorally three times at 5‐day intervals.	12	N/A	2019	2023
Stimotimagene copolymerplasmid	NCT05578820	Advanced‐stage solid tumors	I	N/A	pDNA/PEI– PEG– TAT (PPT)	Ganciclovir	Stimotimagene copolymerplasmid was administered intratumorally once (20 or 40 µg/cm^3^), twice, or three times at 5‐day intervals, based on the trial phase. Ganciclovir was given intravenously twice daily for 15 days in all phases.	21	N/A	2022	2024

Abbreviations: cART, combination antiretroviral therapy; N/A, not available currently; PHPC, high proliferating capacity.

Several clinical trials have evaluated GEN‐1 (IMNN‐001), a lipopolymer‐based gene therapy that delivers IL‐12 pDNA. Plasmids in this formulation are protected from DNases by being condensed with the PEG‐PEI‐cholesterol formulation. PEG also promotes nanoparticle stability within the body. An early phase I trial of IMNN‐001 (NCT00137865) showed elevated levels of IL‐12 and IFN‐γ in peritoneal fluid, indicative of localized immune activation. However, serious adverse events were reported, including abdominal infections at doses exceeding 12 mg/m^2^ with dose‐limiting toxicities [224]. IMNN‐001 showed limited efficacy in treating platinum‐resistant ovarian cancer, with 35% of patients achieving stable disease and a 30% progression‐free survival rate at 6 months [225]. Based on these findings, IMNN‐001 demonstrated poor tolerance and limited efficacy in patients with platinum‐resistant recurrent ovarian cancer. Additionally, in the Phase I/II OVATION 2 trial (NCT03393884), IMNN‐001 combined with neoadjuvant chemotherapy showed a 31% reduction in the risk of death (HR: 0.69) and improved progression‐free survival in advanced ovarian cancer. Further, a Phase II trial (NCT05739981) is underway to reveal the outcomes of platinum‐resistant ovarian cancer when combined with bevacizumab. Additional studies with expanded cohorts are needed to further realize the therapeutic potential of GEN‐1 and leverage its immune‐stimulating properties for optimal efficacy and improved tolerability in ovarian cancer patients.

Extensive studies have demonstrated the high efficacy of PEI as an adjuvant for vaccines, but only a small number of PEI‐based vaccines have been tested in clinical trials. For example, the DermaVir vaccine, composed of pDNA encoding HIV‐1 gag in complex with mannosylated PEI 22 kDa, was designed to specifically target Langerhans cells with uptake through receptor‐mediated endocytosis [226]. Mannosylated PEI was critical for the recognition and uptake of the vaccine by APCs and effectively disrupted endosomes [227] and release of pDNA into the nucleus [228]. In the first clinical trial, DermaVir (NCT00712530) was administered in three different doses to nine HIV‐infected adults (0.1 mg, 0.4 mg, or 0.8 mg pDNA per subject) with no reported treatment‐related adverse events of grade 3 or higher (according to the DAIDS AE Grading Table) [229]. In participants who received the higher dose, HIV‐specific T‐cell precursors with a high proliferation capacity increased significantly compared to untreated subjects. Importantly, HIV RNA levels remained below 50 copies/mL, and CD4+ counts were stable in all participants, regardless of vaccine dose, demonstrating that DermaVir immunization was not associated with detectable viral activation or transient viral blips. T‐cell responses diminished over time but were still detectable up to 48 weeks following vaccination, although reduced proliferation capacity was observed.

The IFx‐Hu2.0 vaccine, containing pDNA encoding Emm55 combined with JetPEI and stabilized with 5% dextrose, was applied to treat advanced nonmelanoma skin cancer [230]. When IFx‐Hu2.0 is delivered intratumorally, Emm55 is expressed in tumor cells by activating innate and adaptive immune systems. An ongoing phase I clinical trial (NCT04160065) has shown that the combination of IFx‐Hu2.0 with anti‐PD‐1 agents, administered weekly intratumoral dosing for up to 3 weeks, is safe and well‐tolerated with no severe treatment‐related adverse events reported [231]. A durable objective response lasting 7–20+ months was also achieved in 71% of patients who received immune checkpoint inhibitors after IFx‐Hu2.0 therapy.

Another Phase I/II clinical trial (NCT00270205) evaluated the safety and efficacy of escalating doses of DermaVir in HIV‐infected adults receiving suppressive combination antiretroviral therapy. Similar to the previous trial, no significant adverse effects were observed compared to the placebo group, with similar incidences of grade 1 and 2 events and no grade 3 adverse events [232]. While no therapeutic effect was evident during the initial 12 weeks, the intermediate‐dose group (0.4 mg) showed limited efficacy with a substantial increase in HIV‐specific precursor T‐cells. However, no significant changes were observed in HIV‐specific antibody levels or CD4^+^ cell counts. Like the first clinical trial, insufficient efficacy prevented support for further investigation, causing DermaVir clinical trials to be discontinued.

The reasons behind ceasing these trials are heterogeneous and not necessarily related to PEI‐related safety concerns. For instance, Phase I of IMNN‐001 (NCT00137865) was discontinued since the maximum tolerated dose was never reached, and successful gene transfer was already proven clinically. After multiple trials (GEN‐1 and IMNN‐001), the formulation made progress to eventually reach Phase III, proving that an early‐phase termination was not indicative of platform failure. On the other hand, MK‐4621/JetPEI trials (NCT03065023 and NCT03739138), delivering a RIG‐I agonist intratumorally via linear PEI alone or combined with pembrolizumab, were abandoned for lack of therapeutic benefit, resulting in zero objective responses as monotherapy and marginal responses as combination therapy. Both trials demonstrated that the PEI carrier itself served as the functional carrier since target engagement was observed by downstream biomarker activation, and no trial was abandoned due to carrier‐related complications.

Collectively, this clinical progress provides important guidance for developing PEI‐based nanoformulations in the future since the safety of chemically modified PEI carriers has now been validated across multiple administration routes, dose levels, and patient populations, addressing a longstanding translational concern rooted in preclinical toxicity data. In spite of this, effective intracellular delivery does not guarantee clinical success, as the therapeutic payload, the disease context, and the treatment setting play equal roles in ensuring clinical success. In the process of progressing EGEN‐001 from Phase I as a monotherapy to a Phase III candidate, the platform has not been redesigned but rather optimized through iterative refinement of the combination partners and the patient population. In this scenario, it is better to maintain a clinically validated PEI delivery platform and refine therapeutic strategies around it than to abandon the carrier after initial functional tests are inconclusive.

Overall, clinical trials involving PEI‐based formulations have shown that PEI is a safe, tolerable, and effective adjuvant and delivery agent for vaccines. PEI‐based systems can also load and deliver therapeutic agents efficiently, particularly when targeting cancer and infectious diseases, due to the ability of PEI to boost recognition and uptake by immune cells. Even though PEI‐based formulations are still in development to overcome challenges associated with cytotoxicity and scalability, PEI holds the potential to advance future therapeutics and vaccines.

## 7 Translational Challenges and Future Perspectives

Despite the remarkable advances outlined earlier, translating PEI‐derived nanoformulations to the bedside remains complicated by multiple interrelated hurdles, comprising chemical, biological, and regulatory aspects. These obstacles include dose‐limiting cytotoxicity, protein corona‐mediated targeting failure [233], off‐target hepatic and splenic accumulation [234], and innate immunogenicity via complement and TLR activation [235]. While there are emerging approaches to mitigate cytotoxicity, as discussed previously, there is a paucity of long‐term data on in vivo toxicology in nonhuman primates, which are a prerequisite for IND approval. The cationic surface charges further trigger serum protein adsorption to form a protein corona, masking surface ligands and abrogating active targeting [236]. Off‐target biodistribution of PEGylated PEI/pDNA polyplexes further complicates these challenges, with nanoparticles preferentially accumulating in hepatic, splenic, and pulmonary tissues following intravenous administration, driven by mononuclear phagocyte system sequestration [237].

Further, lot‐to‐lot consistency of PEI conjugates production, nanoparticle size, and surface charges confers additional complexities on GMP compliance. Recent industrial developments have rendered it feasible to achieve GMP‐compliant PEI platforms. PEIpro‐GMP is an aseptically manufactured PEI reagent introduced by Sartorius/Polyplus that bridges this gap since it is ICH Q7‐compliant. Additionally, PEIpro‐GMP is compliant with Ph. Eur. QC, and full regulatory documentation supporting Investigational New Drug (IND) and Biologics License Application (BLA) filings [69]. This shows that PEI‐based systems could be produced with reliable consistency and regulatory traceability, yet additional specifications must be met in translating from in vitro to in vivo therapeutic delivery. This issue is addressed by in vivo‐JetPEI, a clinically advanced linear PEI derivative that qualifies as a clinical‐grade delivery system, manufactured under cGMP and tested for potency, identity, safety, and purity [238]. Several Phase I and Phase II trials have already investigated this reagent, and preclinical safety data indicated that complexes are colloidally stable for up to 1 month at 4°C and neither pro‐inflammatory cytokine induction, nor elevated liver enzymes. Together, these platforms demonstrate the ability to address the major manufacturing challenges inherent in the clinical application of PEI, such as reproducibility, regulatory compliance, and pharmacopeial standardization; nonetheless, they do not exclude inherent biological obstacles outlined earlier. As of today, PEI‐derived nanoplatforms have advanced beyond early‐phase clinical evaluation, and progressing further calls for standardized in vitro–in vivo correlation models and transparent reporting of failure cases.

In the aftermath of the clinical validation of mRNA vaccines developed for COVID‐19, mRNA has gained prominence as a transformative therapeutic modality. Clinical translation, however, presents several specific challenges. These include protecting the relatively fragile mRNA backbone during charge‐driven complexation, optimizing endosomal escape kinetics relative to siRNA or plasmid DNA for larger RNA cargoes [164], and boosting rather than suppressing antigen expression by controlling PEI‐induced innate immune activation. However, the predominant use of lipid nanocarriers has resulted in logistical and biological challenges, which could be remedied with PEI‐derived nanoformulations. Among these challenges are the ultra‐cold chain demands of current LNP‐mRNA vaccines, such as BNT162b2 (–70°C) and mRNA‐1273 (–20°C), which severely limit their worldwide distribution, particularly in regions lacking adequate cryogenic infrastructure [239, 240].

Further, anaphylaxis triggered by PEGylated lipids has also been reported as a rare but potentially serious safety concern [241]. It is conceivable that PEI‐based mRNA carriers would retain colloidal and functional stability at refrigerated or even ambient temperatures, providing a feasible approach to mRNA vaccines with enhanced thermostability [241, 242]. As previously outlined, biodegradable and charge‐tuned PEI derivatives and stimuli‐responsive platforms serve as a rational design framework for addressing these biocompatibility challenges, and some approaches provide an attractive avenue for scalable and globally deployable mRNA vaccines. Moreover, PEI's inherent adjuvant‐like activity could also augment antigen presentation and innate immune priming without further adjuvants, a feature already employed in preclinical mRNA cancer and infectious disease vaccines [243, 244]. However, a deeper understanding of the immunological responses to PEI is crucial, particularly its interactions with DCs and other APCs. The efficacy of PEI as an adjuvant could also be greatly enhanced when coupled with other polymers, such as PLGA, PEG, and polycaprolactone, as well as other organic and inorganic nanoplatforms. These PEI‐modified formulations are more stable with controlled particle size and sustained antigen release, enhancing immune responses. Theranostic potential and cargo stability could be further enhanced when hybrid PEI systems are fabricated with advanced nanomaterials, such as MXenes, MSNs, QDs, carbon nanotubes, and HNTs. Integrating advanced nanotechnology with functionalization methods can create PEI analogs and systems that are more versatile, safe, and effective for clinical applications. New PEI analogs, such as ultra‐low‐molecular‐weight derivatives and biodegradable analogs, provide platforms for safer, more sustainable, and clinically viable PEI therapies. In addition, biomimetic coatings or functionalization of PEI‐based constructs with PEG molecules makes these systems more biocompatible with immune camouflaging ability. At the same time, modifications with stimuli‐responsive properties can create systems responsive to pH, redox changes, light, or ultrasound that facilitate on‐demand cargo delivery.

Similarly, the PEI potential also extends to CRISPR/Cas genome editing, where delivery technology remains a major translational obstacle [245]. PEI‐based platforms are well‐suited to circumvent the challenges associated with currently widely applied CRISPR/Cas delivery via adeno‐associated virus vectors (AAVs) and lipid nanoparticles. The application of AAVs exhibits limited cargo capacity of 4.7 kb with the potential of eliciting neutralizing immunity [246]. Further, AAVs are raising therapeutic counterproductive concerns, persist as episomal DNA, driving sustained Cas9 expression and off‐target risk even in single‐event editing [247]. On the other hand, PEI platforms provide a nonintegrating, transient expression profile, capable of accommodating the full cargo range of CRISPR components [248]. Preclinical studies using modified PEI variants have demonstrated effective co‐delivery of CRISPR components and achieved gene editing and apoptosis comparable to Lipofectamine 2000 in A549 cells [249]. Nevertheless, it remains technically challenging to deliver heterogeneous Cas9 RNP complexes in a stoichiometric manner [250]. Moreover, nuclear delivery to nondividing cells is inefficient [251], and the competence to tailor transient expression windows to limit off‐target editing is beyond the capabilities of current PEI formulations. Addressing these concerns calls for advanced combinatorial designs of smart PEI platforms that possibly will make it feasible for CRISPR cargo beyond capacity limits to perform with competitive gene editing efficiencies with better immunological tolerance for several doses at lower costs, scalability, and distribution issues [32].

Clinical translation of PEI‐based formulations could benefit from improved designs that reduce cytotoxicity and enhance targeting specificity and nuclear delivery. PEI delivery systems currently lack standardized comparative in vivo studies assessing efficacy and safety against other nonviral delivery systems, particularly when assessing cost‐effectiveness and ease of preparation. Moreover, further consideration should be given to whether PEI modifications offer genuine therapeutic value or introduce unnecessary trade‐offs. Modification of PEI can reduce cytotoxicity and improve efficacy, although unmodified PEI may be effective at lower doses, steering away from complicated and costly synthetic procedures.

Development of methods for synthesizing PEI nanoparticles should be dedicated to optimizing efficiency and scalability while serving specific application demands, such as RNA delivery, vaccine development, and drug delivery. For these purposes, microfluidic‐assisted synthesis provides superior control over the size and shape of produced particles, yielding reproducible, uniform formulations. Following the development of microfluidic Tesla structure chips, PEI nanoparticle preparation continues to improve, producing uniformity, reduced polydispersity indices, and improved encapsulation efficiencies that offer more precise synthesis methods, alternative to current bulk physical mixing methods [252]. Uniform particles suitable for drug and vaccine delivery can also be created with tunable surface properties through electrospraying [253]. Self‐assembled nanoparticles are best suited for drug delivery as they can be designed with tailored properties, such as hydrophobic cores for encapsulating poorly water‐soluble drugs and surface‐responsive properties [254, 255]. LbL assembly can create multifunctional nanoparticles with enhanced cell targeting and endosomal escape functions suitable for RNA and vaccine delivery [96]. For long‐term stability of pharmaceuticals or vaccines, spray‐ or freeze‐drying can be used either as an after‐treatment or for formulation [256, 257]. Optimizing these techniques according to the specific needs of a particular application advances the use of PEI nanoparticles for precision medicine [258].

## 8 Conclusion

In conclusion, PEI is a rising star in drug and gene delivery driven by the knowledge being acquired from increasing research into the design, preparation, and applications of PEI‐based systems. With cationic properties, PEI exhibits adsorption capabilities toward a wide range of therapeutic cargoes. However, PEI‐based formulations often exhibit high cytotoxicity that limits clinical studies and therapies. PEI cytotoxicity is mainly due to its limited biodegradability and high cationic charge, which disrupt cell membranes, induce oxidative stress, and damage mitochondria. Higher molecular weight PEI induces higher cytotoxicity than lower molecular weight PEI, but modifications like PEGylation can mitigate these effects. Notably, chemical derivatization, nanoparticle neutralization, shielding, or targeting have shown promise for reducing cytotoxicity associated with PEI polymers. Further research is needed to understand and mitigate the adverse reactions to PEI to open new avenues for future clinical applications.

## Funding

This work was supported by NHMRC (GNT1162684, GNT2012583 and GNT2028090).

## Conflicts of Interest

The authors declare no conflicts of interest.

## Data Availability

Data sharing not applicable to this article as no datasets were generated or analyzed during the current study.
